# Automatic pose estimation in newborn infants: Lessons from the Baby Grow study

**DOI:** 10.3758/s13428-026-02943-z

**Published:** 2026-03-09

**Authors:** Mohammad Saber Sotoodeh, Ori Ossmy, Georgina Donati, Jazmine Hall, Hannah Rowan, Gillian S. Forrester

**Affiliations:** 1 School of Psychology, University of Sussex, Brighton, UK; 2 Centre for Brain and Cognitive Development and School of Psychological Sciences, Birkbeck, University of London, London, UK; 3 Department of Psychiatry, Warneford Hospital, University of Oxford, Oxford, UK

**Keywords:** Markerless motion tracking, Biomechanics, Motor development, General movement, Computer vision

## Abstract

Advances in computational techniques—particularly machine learning—have expanded opportunities to analyse early infant motor repertoires, especially in naturalistic settings. The aim of this study was to evaluate the strengths, limitations, and performance of state-of-the-art pose estimation algorithms in challenging, home-based video conditions. We analysed 22 videos recorded by parents using mobile phones from eight newborns in the Baby Grow study, at 2, 4, and 8 weeks of age. The videos varied in clothing (common onesie, babygrow, vest), background (grey, black, coloured), lighting (with/without shadows), and camera angles (top, front, bottom). From these, 2,640 frames were extracted and manually annotated to serve as ground truth. We tested demo versions of MediaPipe, OpenPose, PCT, RTMpose, Sapiens, and VitPose, and evaluated performance using object keypoint similarity (OKS), percentage of correct keypoints (PCKh), speed, and accuracy. RTMpose showed the highest overall accuracy, while MediaPipe had the fastest processing speed. However, when balancing speed and accuracy at ratios of 70:30, 50:50, and 30:70, MediaPipe’s speed compensated for its lower accuracy, making it a strong candidate for practical applications. Model performance varied under different environmental conditions, with RTMpose, Sapiens, and VitPose being the most robust. As infant movement research increasingly shifts to real-world environments, selecting appropriate models and ensuring video quality are essential. Our findings show that (1) new models outperform legacy tools like OpenPose, and (2) video context and model selection significantly affect pose estimation accuracy.

**Supplementary Information:**

The online version contains supplementary material available at 10.3758/s13428-026-02943-z.

## Introduction

Motor development during infancy and its subsequent associations have been an important focus for researchers in recent decades, especially for those researching neurodevelopmental conditions. Early movement repertoire (general movements, GMs) has served as reliable screening criteria for the early diagnosis of cerebral palsy (CP) (Einspieler & Prechtl, 2005; Hadders-Algra, 2004), and there is evidence that other neurodevelopmental conditions, such as autism and attention-deficit/hyperactivity disorder (ADHD) (Arabameri & Sotoodeh, 2015; Gao et al., 2023; Wilson et al., 2024), where social and communication features currently act as hallmark symptoms later in development, may show early disruptions to motor behaviour (Wilson et al., 2024). This suggests that reliable assessment and tracking of early motor development are useful not just from a research perspective, but potentially also from clinical and diagnostic perspectives. While clinical and traditional methods (questionnaires, checklists, and motor development test batteries) remain central to motor development assessment, these methods have their limitations, for example, in training, time, detail, and objectivity (Ossmy et al., 2025). Advances in computational techniques, particularly machine learning, are able to address these issues and have expanded research potential in investigating features of infant motor repertoires (Leo et al., 2022; Ossmy et al., 2025; Stagni et al., 2023).

With recent advancements in computer science and sensor technologies, two primary automated methods are now employed to evaluate infant motor movements: two-dimensional (2D) pose detection models based on computer vision (Adde et al., 2018; Marchi et al., 2019; Schroeder et al., 2020) and wearable sensors (Marcroft et al., 2015; Redd et al., 2019). Two-dimensional pose detection stands out in particular due to its markerless nature and reliance on standard RGB video recordings. This allows researchers and clinicians to assess infant movements in more naturalistic and remote settings without the need for intrusive equipment.

Despite significant advances in pose estimation algorithms, OpenPose (Cao et al., 2019, 2021), DeepLabCut (Mathis et al., 2018), and recently MediaPipe (Lugaresi et al., 2019) remain among the most widely used tools in infant motor development research (Gao et al., 2023; Marcroft et al., 2015; Rosales et al., 2024). However, recent developments suggest that newer models may offer improved performance. For instance, VitPose has demonstrated state-of-the-art accuracy in human pose estimation (HPE) using the COCO dataset (Jahn et al., 2025; Xu et al., 2022), and further refinements continue to emerge (see Gama et al., 2025, and Jahn et al., 2025, for an overview of more recent algorithms potentially outperforming existing models).

Nonetheless, a critical limitation persists: current pose estimators are predominantly trained on datasets featuring adults, yet are applied to infants without any adaptation. This is largely due to the absence of large, publicly available infant datasets. The field of infant pose estimation faces significant challenges related to data sharing, which hinder the development of robust, generalisable models (Marschik et al., 2023). As a result, infant-specific models (Cao et al., 2022; Huang et al., 2021; Moccia et al., 2020; Soualmi et al., 2024; Yin et al., 2024) are typically trained and validated on limited, siloed clinical datasets. These models have yet to be systematically compared using a common benchmark dataset (Jahn et al., 2025) and may not be representative of the variation of any given condition.

While comparative studies of model architectures exist (Hesse et al., 2019; Soualmi et al., 2024; Yin et al., 2024), they are confined to the same training data, raising questions about their ability to generalise to new datasets. Recently, Jahn et al. (2025) showed that VitPose outperformed infant-specific models in a well-controlled laboratory setting. Whether current HPE models retain their performance across challenging conditions remains an open and important question. This is particularly important for studies involving recordings in natural environments such as the infant’s home, where researchers have less control over the videos (different recording angles, quality, and levels of contrast). Because this is a fast-paced area of advancing technology, we focus on established and well-tested pose detection in 2D videos to demonstrate the strengths, weaknesses, and capabilities of these state-of-the-art algorithms for evaluating infant pose detection. We provide key terminology in HPE that will be used throughout this paper.

### Top-down versus bottom-up paradigms in HPE

HPE models utilise deep learning methods to extract and represent features from video/image, with body/limb detection quality depending heavily on the model architecture. Among the most common HPE frameworks are top-down (Liu et al., 2021; Sun et al., 2019; Wei et al., 2016) and bottom-up (Jin et al., 2020; Luo et al., 2021; Wei et al., 2020) approaches. Top-down frameworks first detect human bounding boxes within a frame and then perform pose estimation for each box. Conversely, bottom-up frameworks locate body keypoints across the image and then group them into distinct person instances. Within each approach, there are various subtypes: top-down methods include regression-based, heatmap-based, video-based, and model-compressing methods, while bottom-up methods are categorised into one-stage (Geng et al., 2021; Nie et al., 2019) and two-stage methods (Cao et al., 2017; Kreiss et al., 2019). For an in-depth discussion on these subtypes, see the work of Chen et al. (2023) and Lan et al. (2023).

### Datasets

Annotated datasets are essential resources for training deep learning models. These are data that have been carefully labelled or categorised, making it easier for models to learn specific patterns and relationships. For example, in a dataset of images, annotations might indicate the location and type of objects in each picture. By using these consistent and standardised datasets, researchers can train models to perform tasks such as image recognition or HPE. Furthermore, annotated datasets provide a shared foundation for comparing model performance. These datasets typically vary in the number of images, diversity of humans in context, and the way they label human body parts. Generally, the greater the number of images and diversity of humans in context, the greater the model accuracy; however, it can simultaneously negatively affect the speed of the model. Here, we review commonly used datasets for full-body HPE:

- **COCO**: The Microsoft Common Objects in Context (COCO) dataset (Lin et al., 2014) is one of the most widely used datasets in HPE, containing over 200,000 images and 250,000 person instances labelled with keypoints at various scales. COCO provides annotations with over 150,000 individuals and 1.7 million keypoints across 17 anatomical keypoints (e.g., nose and left/right eyes, ears, shoulders, elbows, wrists, hips, knees, and ankles) (https://cocodataset.org/#keypoints-2017).
- **MPII**: The Max Planck Institute for Informatics (MPII) dataset (Andriluka et al., 2014) is curated based on a taxonomy of over 800 human activities including recreational, occupational, and household settings. MPII captures a broader spectrum of human movement compared to other datasets (http://human-pose.mpi-inf.mpg.de/). Models using MPII generally provide 13 keypoints, including the head, shoulders, elbows, wrists, hips, knees, and ankles.
- **BlazePose**: Developed by Google, BlazePose consists of 60,000 images with single or multiple people in typical postures and 25,000 images of individuals performing fitness exercises (Bazarevsky, 2020). Although not publicly available, BlazePose is used in MediaPipe solutions (Lugaresi et al., 2019) and provides 33 different keypoints including a higher number of keypoints on hands, feet, and face.

Importantly, none of these datasets focus specifically on infants or young children. Infants’ body size and scale (e.g. relative proportion of arm to body length) are different from adults; they usually appear in different clothing from adults and are usually in the supine position, which makes their motor repertoires different from those of adults (Gama et al., 2025). In this study, we chose COCO dataset for the following reasons:

1. All selected models have pre-trained models with COCO or can provide keypoints according to the COCO keypoints list, which makes a fair comparison between them.
2. In the early months after birth, most newborn movements are gross, and clinicians and researchers are less focused on fine motor skills; therefore, COCO keypoints provide enough information for this time point.
3. The body portion and limb size (hands and feet) are smaller in newborns and make their automatic detection less reliable.

### Accuracy, speed, and efficiency

Accuracy remains the most essential metric for evaluating different HPE methods. Average precision (AP), based on object keypoint similarity (OKS), measures the similarity between detected and Ground-Truth (GT) keypoints (Lin et al., 2014). The AP score for each keypoint at various OKS thresholds is noted as AP@N. The percentage of correct keypoints (PCK) is another commonly used evaluation metric in pose estimation tasks. It measures how many predicted keypoints fall within a certain threshold distance from the GTkeypoints, relative to the size of the detected person (Zhang et al., 2025). In this study, we reported calculated PCKh@.5 for all models.

Speed, generally measured by the number of frames a model can process per second, is another critical factor, especially in high-frame-rate scenarios. The trade-off between speed and accuracy is common in these analyses. To aid in the understanding of the performance of the different models and simplify model selection for future researchers, we introduced the efficiency metric, which is a calculation of speed and accuracy weights in different ratios (70:30, 50:50 and 30:70).

As motor research moves into more naturalistic and uncontrolled settings facilitated by technology, new challenges are introduced in reliably analysing such data. Furthermore, as is often the case, research tools are designed with adults in mind and later applied to infants with varying degrees of success. In order to guide researchers in this exciting new field of infant research, this study aims to evaluate the existing algorithms in pose estimation in order to assess which deals best with non-optimal data (e.g. home videos with challenging features) and with varying infant statures and movement profiles.

The aims of this study were:

1. To evaluate the accuracy, speed, and efficiency of recent HPE models on infant movement in varied and challenging conditions.
2. To identify factors affecting model performance and provide guidance for researchers studying infant movements.

For evaluation, we chose MediaPipe (Lugaresi et al., 2019), OpenPose (Cao et al., 2019, 2021), PCT (Gao et al., 2023), RTMpose (Jiang et al., 2023), VitPose (Xu et al., 2022), and Sapiens (Khirodkar et al., 2024) because they are either (1) the most commonly used by researchers (OpenPose) or (2) growing in popularity (MediaPipe), or (3) show promising accuracy in published studies (PCT, RTMpose, Sapiens, and VitPose). A brief overview of each method is available in supplementary file S1. Based on previous studies, we predict the highest speed for MediaPipe and RTMpose, the best performance for PCT in complex positions (since it is supposed to predict the keypoint positions in occluded conditions), and the most accurate detection by VitPose and Sapiens, since they both have very similar datasets but different estimations and pre-trained models.

## Methods

### Participants and videos

**Fig. 1:**
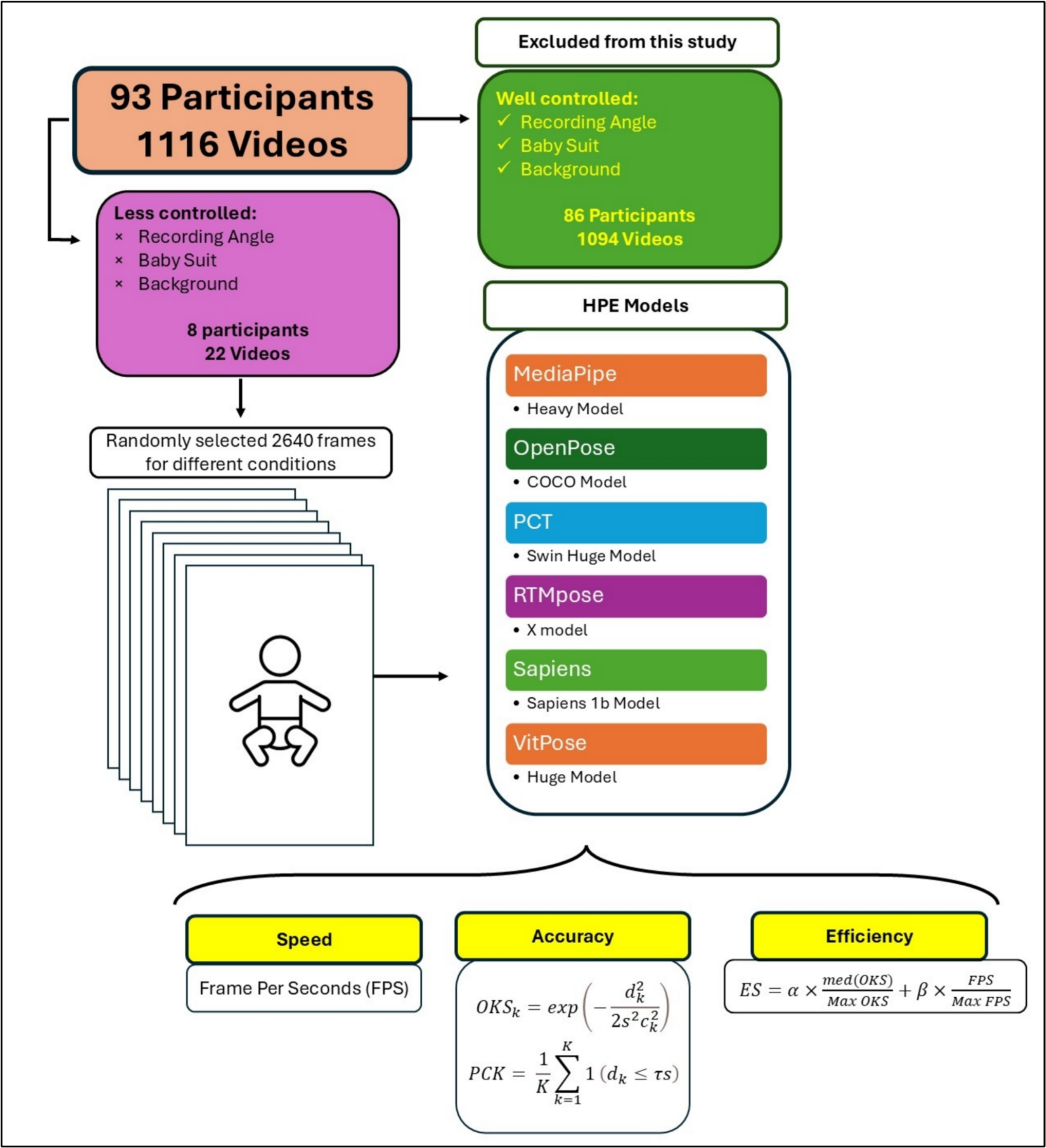
Schematic of this study, starting from top left (video collection) to bottom right (measures)

**Fig. 2:**
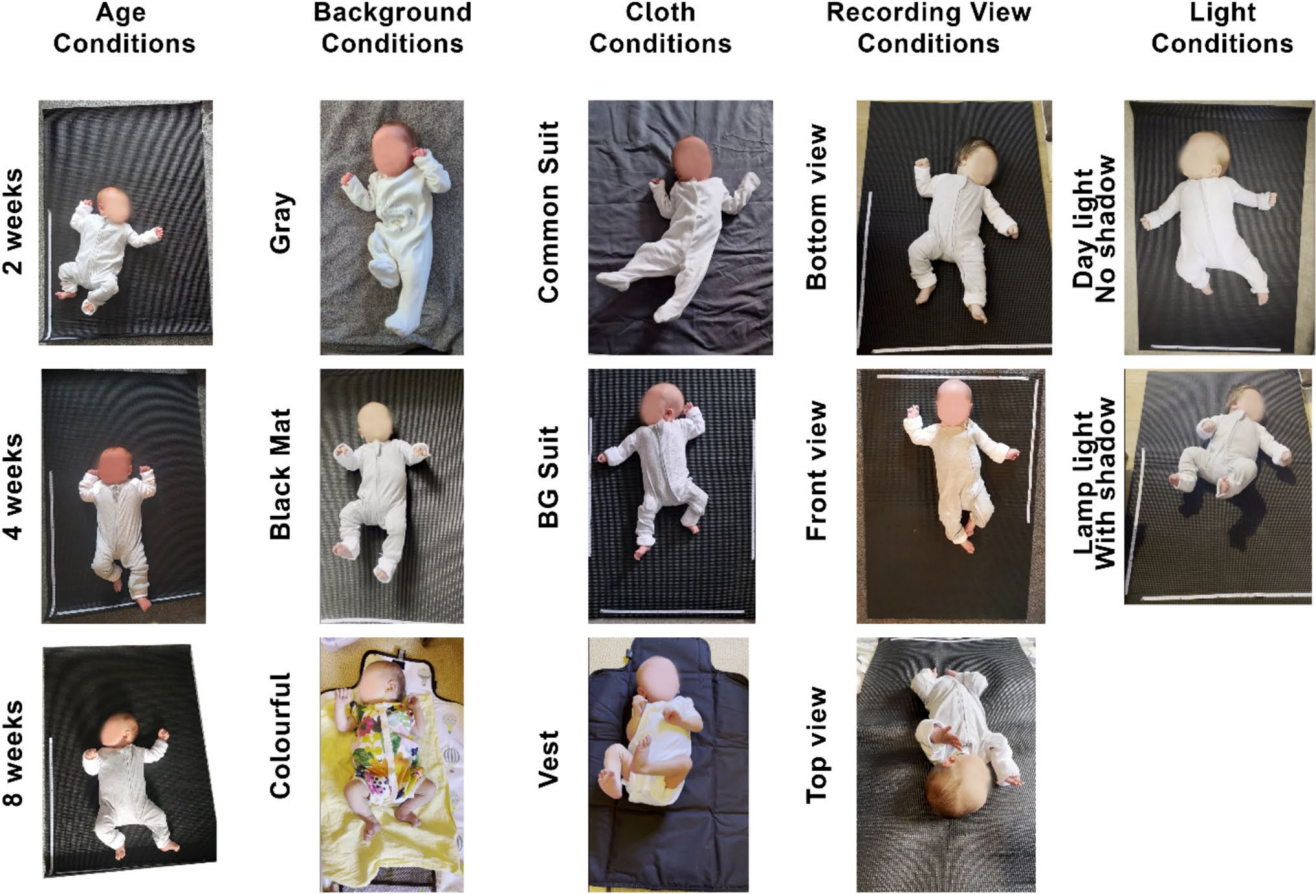
Different challenging conditions to test different HPE models

For this study, we chose 22 videos from eight newborn infants (Table 1) who participated in the Baby Grow study at different ages (2, 4, and 8 weeks). Baby Grow is a longitudinal study (currently ongoing) designed to evaluate early motor behaviours in children at a higher and lower likelihood of neurodevelopmental conditions (autism and ADHD) using weekly home recorded videos and motion sensors. We selected videos with different clothing conditions (common onesie, babygrow [BG], and vest), different background conditions (grey, black, and coloured background), different light conditions (daylight with no shadow, lamp light with shadow), and different recording angles (bottom, front, and top recording views). In order to maximise the quality and usability of the video recordings, participants were sent a BG baby suit, phone-holder, and instructions in an attempt to control for factors such as baby’s clothes, background, recording angle, and light (please see open access data repository for the full instruction manual and further information). Despite these efforts, there were cases (mostly in early recruited participants) where we received videos which included large differences in these variables. Rather than exclude these valuable videos from the study, we decided to test different HPE models to find the most efficient one in overcoming these challenges (Fig. 1). The study was approved by the ethical committee of the University of Sussex in accordance with the ethical standards as laid down in the 1964 Declaration of Helsinki (Reference Code ER/GF235). We checked all videos (*N* = 1,116, from 93 participants) and categorised them in different conditions based on their quality (baby’s clothes, background, recording angle, and light). To make the comparisons fair, we then chose babies with the most variation in the recording (except for one): specifically, we chose three videos at different age points (t 2, 4, and 8 weeks old) from seven different participants in different conditions (Fig. 2), yielding 21 videos, and one video from a baby (MM001) which was part of our pilot study. Since the videos contain sensitive participant information, they are unavailable for public access, but we provided selected frames in this study in the Figshare repository (https://figshare.com/s/d2f4c12f77a5734ab553). From each video, we chose 120 frames with a constant distance between them (see procedure section for more information), which resulted in 2,640 frames overall (see Fig. 2 for examples of video conditions).

**Table 1:** Demographic information for each participant

Participant ID	BG001	BG002	BG003	BG008	BG009	BG014	BG016	MM001
Sex (male, female)	M	F	F	M	M	F	F	F
Gestational age (weeks)	38	39	38	39	40	40	41	41
Gestational weight (kg)	3.93	3.54	2.72	2.88	4.08	2.95	3.5	3.35
Medical condition	No	No	No	No	No	No	No	No

### Procedure

After selecting videos that represented the full range of challenging conditions, a two minute segment from each video was selected by a clinically trained general movement assessor that demonstrated optimal movement activity. The following equation was then used to extract all the frames from the video segments for human and HPE annotations.

$$\begin{array}{l}interval= \frac{en{d}_{time}-star{t}_{time}}{fra{me}_{count}}\\ frame\, time=star{t}_{time}+(interval\times fram{e}_{count})\end{array}$$

Following the extraction of the selected frames, a coder (with a PhD degree in motor behaviour and HPE expertise) annotated different keypoints (nose, eyes, shoulders, elbows, wrists, hips, knees, and ankles) on each frame using the Computer Vision Annotation Tool (CVAT: https://github.com/cvat-ai/cvat). The positions of each keypoint on the *x* and *y* axes for each frame were extracted as the GT for each frame. To test the reliability of our GT, we asked two independent researchers (coder 1 and coder 2) to annotate 10% of frames in each video (11 videos per coder). Then we calculated the inter-class correlation between the GT and each coder for each keypoint. For all keypoints, the inter-rater reliability was excellent (see supplementary file S2). The highest ICC between the GT and coder 1 was for eyes on both the *x* and *y* axes (ICC1 =.999, *p* <.0001, CI_95%_ = 1–1), and the lowest ICC was for right_knee_y (ICC1 =.989, *p* <.0001, CI_95%_ =.99–.99). The highest ICC between GT and coder 2 was again for eyes (ICC1 =.999, *p* <.0001, CI_95%_ = 1–1), and the lowest ICC was for the right_hip_y (ICC1 =.978, *p* <.0001, CI_95%_ =.97–.98).

#### HPE methods and settings

Different HPE methods have different settings and running options. To ensure correct analysis and replication for future studies, we show the different settings we used for each method in Table 2.

**Table 2:** Description of different models and settings

Method	Version	Model	Approach	Default input	Adjustment	Source code	Default output
MediaPipe	0.10.9	Heavy	Top-down	Image	Yes, modified the main code to load from video files and save the detections as a CSV file	Jupyter Notebook	**Image**
OpenPose	1.7.0 (GPU release)	COCO	Bottom-up	Video, image	No	Demo	JSON and AVI
PCT	NA	swin_huge	Top-down	Image	Yes, modified the main code to save the detections as a csv file	Demo	Json and pdf
RTMpose	NA	X	Top-down	Image and video	No	Demo	Json, video
Sapiens	NA	Sapiens-1B^a^	Top-down	Image	No	Demo	Json, image
VitPose	NA	Huge	Top-down	Image and video	No	Demo	Json, image

^a^There is a pre-trained model with 2 billion data points (Sapiens-2B), but due to a lack of GPU capacity and a time-consuming process, we used the 1B model

For each method, we used the highest-parameter models with MS COCO training, except for Sapiens, whose largest model was incompatible with our PC and MediaPipe, which has its own dataset. COCO was chosen because it provided the main keypoints for infant studies and also made our comparison fair between different models. We also used the demo files provided by the developers, with small modifications. Some modifications were made to the demo code for PCT and Sapiens to achieve our desired output (frames per second). We chose the demos because we wanted to make them replicable for future researchers with lower programming and coding skills.

### Metrics and measures

#### Object keypoint similarity (OKS)

OKS is a common metric for validating detections in HPE studies (Ruggero Ronchi & Perona, 2017). This metric is calculated according to the predicted (p) keypoint coordination by each model, in each frame, and the corresponding keypoint in the GT. In order to evaluate each HPE method’s performance, we assigned the confidence score recommended by MS COCO to the GT (Lin et al., 2014; Ruggero Ronchi & Perona, 2017). We first calculated the keypoint Euclidean distance (*d*_k_), then the keypoint similarity (*k*_s_) and OKS for each keypoint. The OKS was calculated for shoulders, elbows, wrists, hips, knees, and ankles as main joints. Since eyes have an important role in most infant studies and model structures (like MediaPipe), we considered eyes in our analysis as well. The following equation was used for the calculation:

$${OKS}_{k}=exp\left(-\frac{{d}_{k}^{2}}{2{s}^{2}{c}_{k}^{2}}\right)$$

where

$$\begin{array}{lc}{d}_{k}=\Vert {P}_{k}^{gt}-{P}_{k}^{det}\Vert \\ s=\sqrt{bbox\_area}\\ {c}_{k}=COCO\ coefficient \left(\sigma\right)\ for\ keypoint\ k\\ overall_{OKS}= \frac{1}{K}\sum\limits_{k=1}^{K}{KS}_{k}\end{array}$$

Following the calculation of OKS, we calculated the average precision (AP). In HPE, AP is the percentage of detections placed under the precision–recall curve, where a higher value represents better precision (Gama et al., 2025; Lin et al., 2014).

#### PCKh (percentage of correct keypoints)

We also calculated the percentage of correct keypoints for all detections as either correct or incorrect. We set a keypoint as “correct” if the distance

$${d}_{k}\le \tau .s$$

where, $s=\sqrt{bbox\_area}$ and $\tau =pck\_threshold$. In this calculation, we set the PCKh at 5% of the distance between the two eyes.

$$PCK= \frac{1}{K}\sum_{k=1}^{K}1\left({d}_{k}\le \tau s\right)$$

#### Missing and redundant detection rates

Missing and extra detection rates are other important measures showing HPE model performance. For each video, we calculated the percentage of frames with missing detections and frames with more than one person detection.

### Processing speed

Another key factor in computational analysis is the processing speed. For this we calculated the speed for each model in terms of the number of frames each model processed per second (FPS).

#### Efficiency measurement

The trade-off between speed and accuracy is a common phenomenon and makes the model selection challenging for researchers, especially when the speed and/or accuracy of different models is close (Chen & Ran, 2019). Here, we introduced the efficiency score (ES) as a factor in our analysis, which is based on the weighted accuracy and speed measures to create a new measure of model efficiency:

$$ES=\alpha \times \frac{med(OKS)}{Max\ OKS}+\beta \times \frac{FPS} {Max\ FPS}$$

Here

*ES*: is the efficiency score,
α: is the weighting factor for the OKS (which we considered as 70, 50, and 30),
*OKS*: is the object keypoint similarity, and
*Β*: is the weighting factor for speed (which we considered as 30, 50, and 70).

Please note that the weighting we chose in this equation was based on our choice to prioritise accuracy over speed, based on multi-objective optimisation (Deb, 2011).

#### Hardware and operating system

All analyses were performed on a Dell PC with an Intel® Xeon® CPU E5-1650 v4, 3.60 GHz, with 64 GB physical RAM, and an NVIDIA Quadro 5000 GPU with 8 GB memory. The operating system was Windows 10 Enterprise version 22H2. Analyses were performed on Windows Subsystem for Linux (version: 2.3.24.0), which is a Microsoft solution for accessing the Linux terminal in the Windows environment. During the process, all other applications were closed in order to use the complete capacity of the system for the HPE detections.

### Statistical analysis

Since the results of the Kolmogrov–Smirnov test for normality showed non-normal distribution of the data in all models (*p* <.001), we used non-parametric tests for comparing these metrics in the models. For multiple dependent comparisons, we used the Kruskal–Wallis test, and for pairwise comparisons we used the median-ranked Wilcoxon test. A Bonferroni correction was applied to adjust *p* values for multiple pair comparisons.

### Data sharing

All data used in this study including GT, model detections, codes, main statistics, and further statistical analysis with details and plots are available for download from our repository at (https://figshare.com/s/d2f4c12f77a5734ab553) and with this DOI: 10.25377/sussex.28070360.

## Results

### Test of accuracy

#### Average precision

Average precision (AP) provides a comprehensive viewpoint on the performance of different methods. Table 3 shows that RTMpose has the highest AP scores, followed by VitPose, Sapiens, PCT, OpenPose, and MediaPipe.

**Table 3:** Average precision for different methods

Method	AP50	AP75	AP90	mAP	Rank
MediaPipe	.667 ±.296	.025 ±.055	0 ± 0	.196 ±.121	6
OpenPose	.659 ±.352	.075 ±.131	0 ± 0	.162 ±.219	5
PCT	.867 ±.236	.397 ±.328	.04 ±.011	.43 ± .207	4
RTMpose	.948 ±.148	.584 ±.342	.025 ±.05	.55 ±.188	1
Sapiens	.92 ±.19	.493 ±.38	.024 ±.059	.495 ±.215	3
VitPose	.931 ±.184	.496 ±.356	.009 ±.019	.5 ±.189	2

#### Object Keypoint Similarity (OKS)

Although the AP is a common metric in HPE studies, considering the non-normal distribution in the calculated OKS, we demonstrate how well different methods work in comparison with each other in general and under specific conditions (Fig. 2).

#### Overall performance

**Fig. 3:**
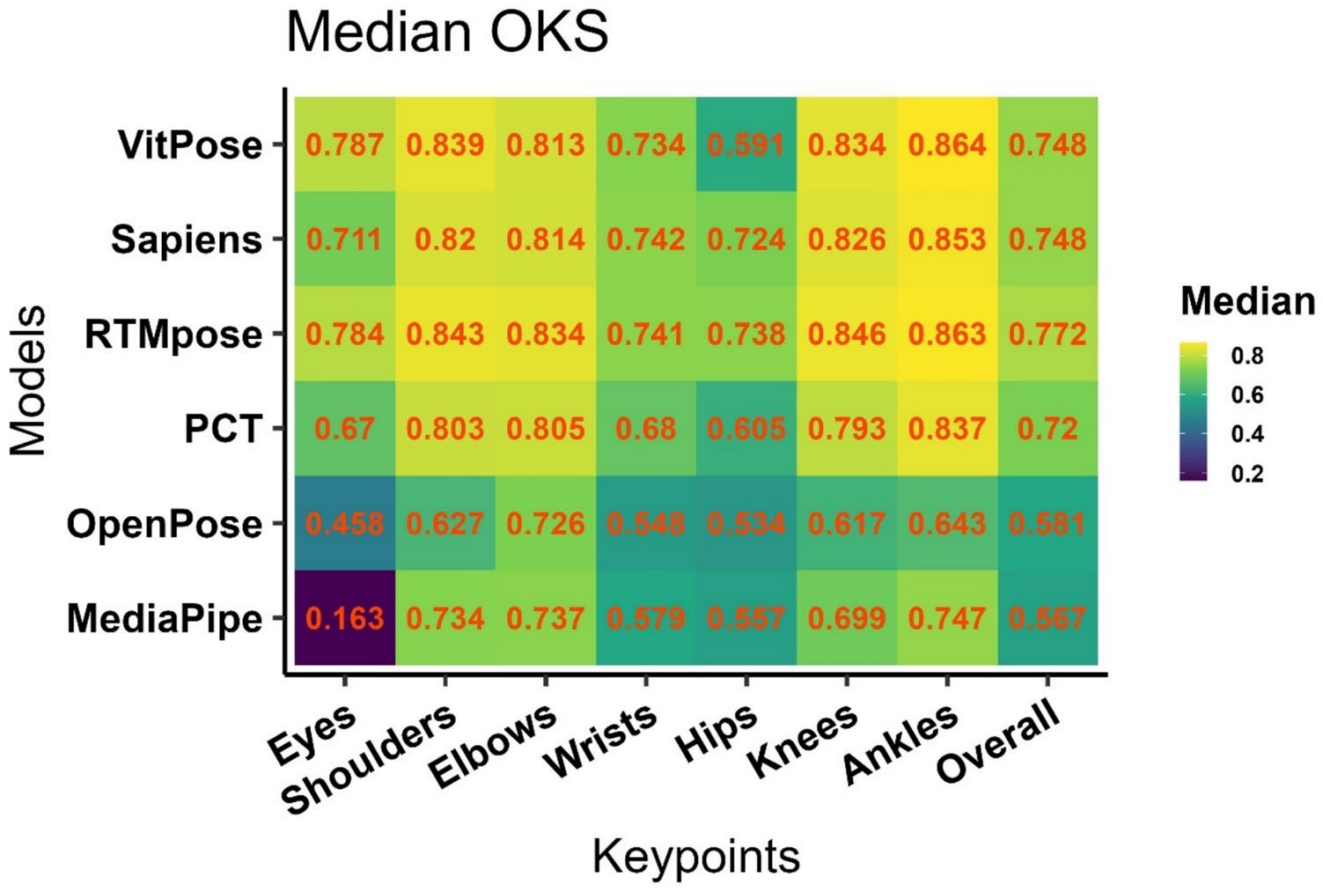
Object keypoint similarity (OKS) for different keypoints and models

**Fig. 4:**
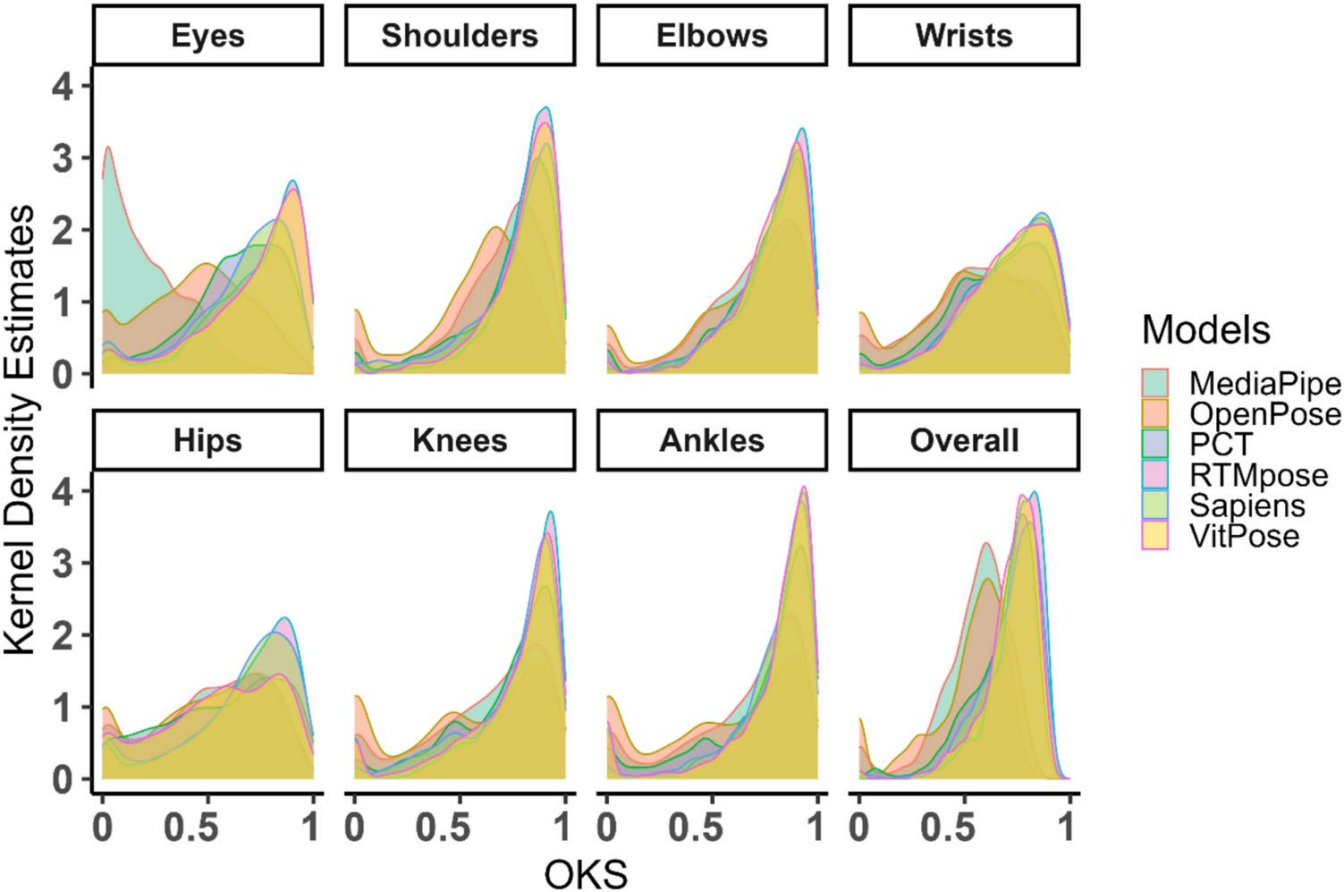
Kernel density estimates (KDEs) for different models across various body parts. RTMpose, VitPose, and Sapiens are the top-performing models across all keypoints and overall performance. MediaPipe and OpenPose have the broadest and least consistent distributions, indicating that they may be less suitable for precise pose estimation tasks in infants. This shows how model choice can significantly impact the accuracy and reliability of pose estimation results for specific body parts

**Fig. 5:**
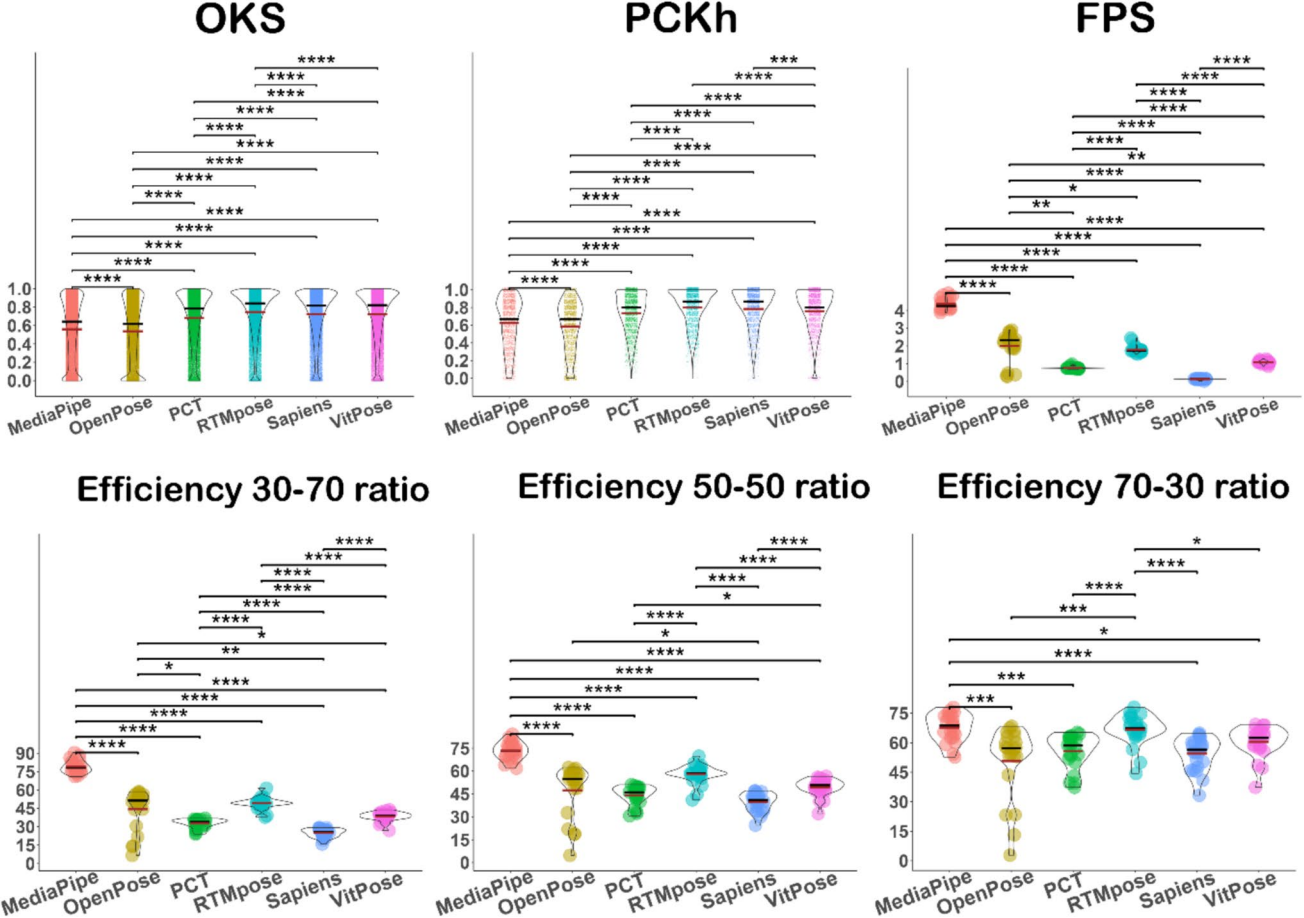
Accuracy (OKS and PCKh), speed (FPS), and efficiency for all methods

Considering all detections (in 2,640 frames), the results of the Kruskal–Wallis test (*H* = 13,468.7, *p* <.0001) showed that the effect of the model on the calculated OKS was significant. In Fig. 5, OKS shows the pairwise comparisons, with the best performance for RTMpose (mean ± *SD* =.746 ±.124, median =.772), then VitPose (mean ± *SD* =.717 ±.132, median =.748), Sapiens (mean ± *SD* =.715 ±.141, median =.748), PCT (mean ± *SD* =.678 ±.153, median =.72), OpenPose (mean ± *SD* =.529 ±.202, median =.58), and MediaPipe (mean ± *SD* =.534 ±.16, median =.567). However, the performance of each method in the detection of different keypoints was different. As shown in Fig. 3, hips are the most difficult keypoints for detection by all models, in addition to the eyes for MediaPipe and OpenPose. Figure 4 shows kernel density estimates of each variable for the entire dataset, divided by model (indicated by colours). Each subplot corresponds to a specific keypoint, and the curves represent the distributions of the OKS scores for various HPE models. For eyes, the OKS scores for MediaPipe are shifted to the left, indicating lower accuracy, whereas RTMpose and VitPose are sharply concentrated near 1, implying high accuracy for these models. For shoulders, elbows, and wrists, the OKS distributions for MediaPipe and OpenPose are broader, indicating more variability or lower accuracy. RTMpose, VitPose, Sapiens, and PCT have narrower distributions peaking closer to 1, suggesting superior performance. For hips, knees, and ankles, similar trends are observed, where RTMpose, VitPose, Sapiens, and PCT consistently outperform the others, with sharp peaks near 1. For overall OKS, RTMpose has the narrowest peak, centred very close to 1, suggesting the highest overall performance.

#### PCKh (percentage of correct keypoints)

PCKh provides greater insight with respect to accuracy. To test the main effect of models on PCKh, a Kruskal–Wallis test was used and showed a significant difference between models (*H* = 1,469.737, *p* <.0001). In Fig. 5, PCKh shows the pairwise comparisons. Similar to the OKS, the highest PCKh was for RTMpose (mean ± *SD* =.801 ±.197, median =.867) and Sapiens (mean ± *SD* =.783 ±.215, median =.867), followed by VitPose (mean ± *SD* =.759 ±.226, median =.8), PCT (mean ± *SD* =.736 ±.219, median =.8), MediaPipe (mean ± *SD* =.623 ±.283, median =.667), and OpenPose (mean ± *SD* =.586 ±.28, median =.667).

#### Missing and redundant detection rates

Missing and redundant detection rates tell us more about the capability of different models in detecting people in the frames. In some cases, HPE models may miss detecting a person or detect more people in each frame than actually exist, which makes post-processing more complicated. Table 4 shows that OpenPose has the highest missing rate, followed by MediaPipe and PCT, whereas RTMpose, Sapiens, and VitPose have no missing frames. On the other hand, RTMpose, PCT, VitPose, and Sapiens had redundant (false) detections.

**Table 4:** Missing/redundant detections in different models

	MediaPipe	OpenPose	PCT	RTMpose	Sapiens	VitPose
Missing rate	.009 ±.018	.033 ±.114	.007 ±.027	0	0	0
Redundant detection	0	0	.08 ±.092	.078 ± .116	.041 ±.105	.07 ±.117

### Test of speed

#### Processing speed

The results of the Kruskal–Wallis test showed that the processing speed is significantly different between models (*H* = 95.205, *p* <.001). In Fig. 5, FPS shows the pairwise comparisons. Although the MediaPipe method was the least accurate, it was the fastest method (mean ± *SD* = 4.353 ±.312, median = 4.241), followed by OpenPose (mean ± *SD* = 1.993 ±.839, median = 2.316), RTMpose (mean ± *SD* = 1.79 ±.215, median = 1.737), VitPose (mean ± *SD* = 1.08 ±.091, median = 1.084), PCT (mean ± *SD* =.762 ±.062, median =.787), and Sapiens (mean ± *SD* =.131 ±.032, median =.14).

### Test of efficiency

#### Efficiency scale (accuracy/speed)

The results of the Kruskal–Wallis test showed a significant difference between models in the efficiency score for the 70:30 ratio (*H* = 45.872, *p* <.001), 50:50 ratio (*H* = 89.029, *p* <.001), and 70:30 ratio (*H* = 100.595, *p* <.0001). In Fig. 5, the bottom rows show the pairwise comparison for these ratios, and Table 5 shows that the processing speed of MediaPipe compensated its low accuracy level, achieving the highest efficiency score for all ratios.

**Table 5:** Efficiency of the models in different ratios

	70 (OKS):30 (FPS)		50 (OKS):50 (FPS)		30 (OKS):70 (FPS)	
	Mean (*SD*)	Median	Mean (*SD*)	Median	Mean (*SD*)	Median
MediaPipe	67.69 ± 7.13	68.664	73.35 ± 5.82	73.312	79.01 ± 5.28	78.109
OpenPose	50.63 ± 18.7	57.14	47.46 ± 17.11	54.801	44.28 ± 16.38	51.301
PCT	55.66 ± 8.76	58.546	44.13 ± 6.25	46.112	32.60 ± 3.78	33.93
RTMpose	66.65 ± 8.03	67.504	57.89 ± 6.31	58.574	49.12 ± 4.94	49.136
Sapiens	54.52 ± 8.44	56.43	39.69 ± 6.01	41.085	24.86 ± 3.6	25.704
VitPose	60.40 ± 7.88	62.412	49.37 ± 5.81	50.711	38.35 ± 3.83	39.01

#### Test of challenging conditions

Analysing newborn babies in naturalistic conditions (such as their home) has unique challenges. To illustrate the accuracy of various methods under these challenging conditions, we show the accuracy of HPE methods for different ages, clothes, and background conditions, recording angles, and light conditions. We report OKS results, since it has been shown to be the gold standard for the COCO database (Jin et al., 2020; Lin et al., 2014). Also, detailed comparison plots for each condition can be found in the supplementary file S3.

#### Tests of age

**Fig. 6:**
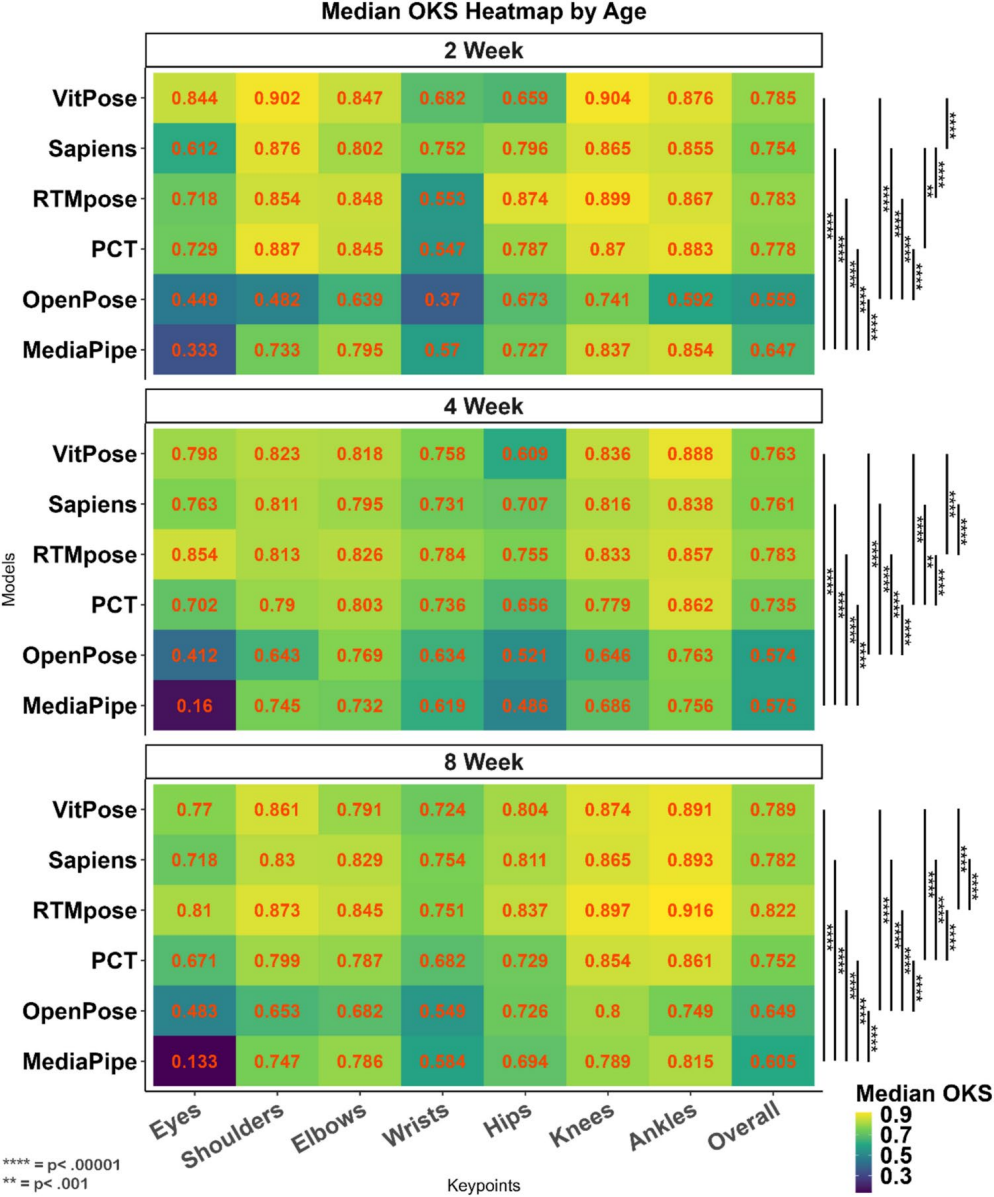
Heat maps for the accuracy of different methods (OKS) for babies at 2 (**top**), 4 (**middle**), and 8 (**bottom**) weeks of age. Pairwise comparison between different models on overall OKS was conducted using the Wilcoxon median-rank test

Most HPE methods predict keypoints based on the backbone skeleton calculations in the model. Since the relative body size in newborn babies is different from that of adults, on which HPE models have been trained, we considered age as an important factor, especially in the early weeks when a baby's body proportions are very different from that of adults and its growth is fast (Villar et al., 2014). The results of the Kruskal–Wallis test (Table 6) showed that the differences between models are significant at different ages (*p* <.001). As Table 6 reveals, the RTMpose method has the best performance at 4 and 8 weeks, and VitPose has the best performance at 2 weeks, while MediaPipe has the least accurate detections at all age points. Figure 6 reveals that in all ages, the eyes, wrists, and hips are the most challenging keypoints for all methods.

**Table 6:** The effect of age on the accuracy (OKS) of different models

Age (weeks)	Model	Mean (*SD*)	Median	Rank (median)	*H*	p*
2	MediaPipe	.635 ±.119	.647	5	752.658	<.0001
	OpenPose	.527 ±.142	.559	6		
	PCT	.768 ±.069	.778	3		
	RTMpose	.774 ±.08	.783	2		
	Sapiens	.752 ±.058	.754	4		
	**VitPose**	**.784** ± **.054**	**.785**	**1**		
4	MediaPipe	.536 ±.162	.575	4	1,780.305	<.0001
	OpenPose	.572 ±.114	.574	5		
	PCT	.72 ±.094	.734	3		
	**RTMpose**	**.774** ± **.087**	**.783**	**1**		
	Sapiens	.733 ±.121	.761	2		
	VitPose	.75 ±.085	.761	2		
8	MediaPipe	.57 ±.163	.605	6	1,457.782	<.0001
	OpenPose	.555 ±.265	.648	5		
	PCT	.69 ±.185	.752	4		
	**RTMpose**	**.804** ± **.078**	**.822**	**1**		
	Sapiens	.741 ±.161	.781	3		
	VitPose	.771 ±.091	.788	2		

*Results of the Kruskal–Wallis test

#### Tests of background and clothing

**Fig. 7:**
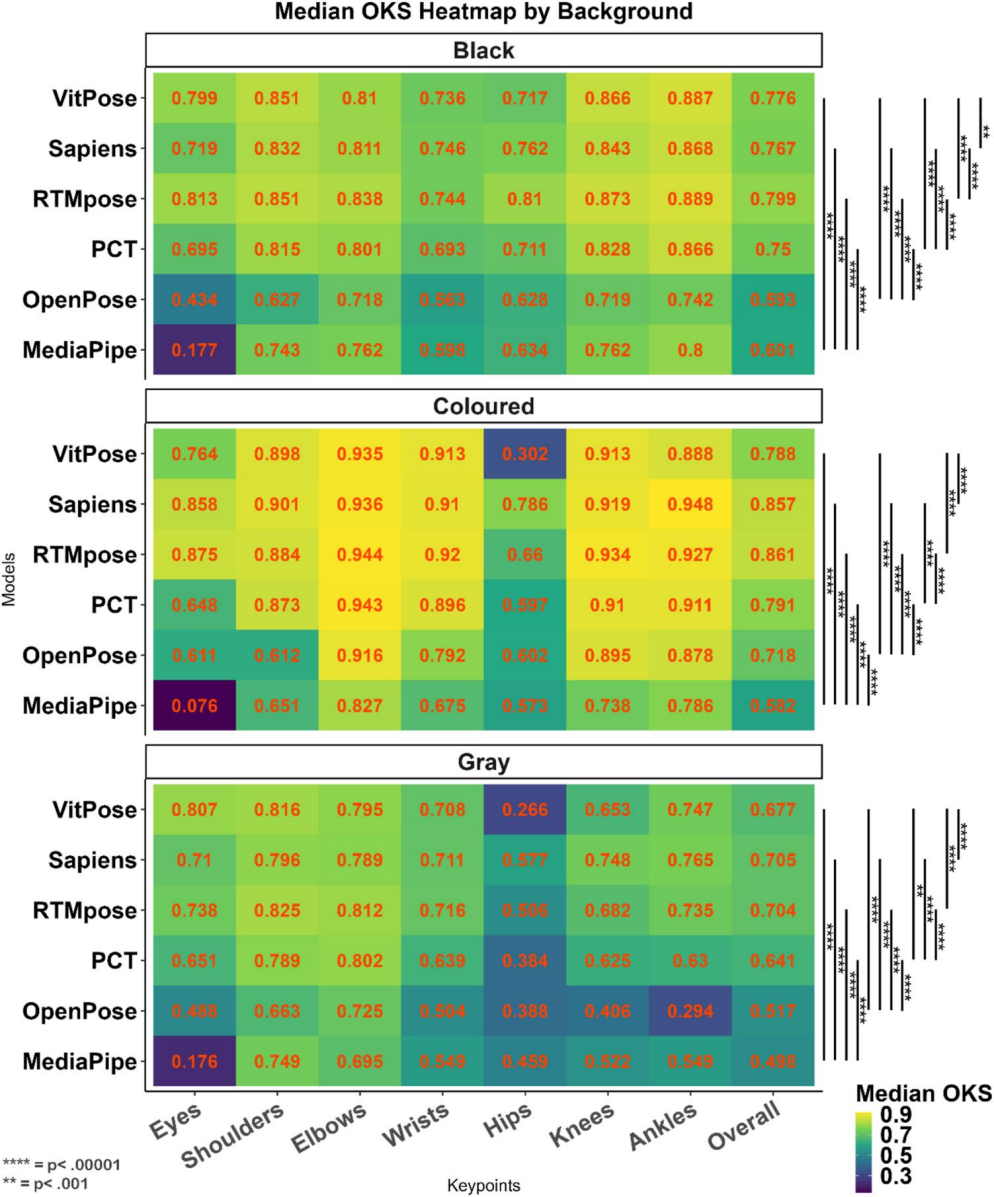
Heat maps for the accuracy of different methods for black (**top**), coloured (**middle**), and grey (**bottom**) backgrounds and keypoints. Pairwise comparison between different models on overall OKS was conducted using the Wilcoxon median-rank test

The results of the Kruskal–Wallis test (Table 7) showed significant differences among models for different backgrounds (*p* <.001). Our results indicated that RTMpose and Sapiens performed best in different background conditions. The complexity of background discrimination is presented well by the RTMpose performance in the coloured and black backgrounds, which was superior to all other methods. Figure 7 shows the performance of all methods and pairwise comparisons for the detection of the keypoints across different background conditions.

**Table 7:** The effect of background on the accuracy (OKS) of different methods

Background condition	Method	Mean (*SD*)	Median	Rank (median)	*H*	p*
Black	MediaPipe	.565 ±.16	.6	5	3,785.893	<.001
	OpenPose	.558 ±.189	.593	6		
	PCT	.714 ±.141	.75	4		
	**RTMpose**	**.787 ±.084**	**.798**	**1**		
	Sapiens	.739 ±.133	.767	3		
	VitPose	.764 ±.085	.775	2		
Coloured	MediaPipe	.562 ±.11	.582	6	477.242	<.001
	OpenPose	.701 ±.083	.718	5		
	PCT	.782 ±.084	.791	3		
	**RTMpose**	**.844 ±.077**	**.861**	**1**		
	Sapiens	.851 ±.048	.857	2		
	VitPose	.775 ±.077	.787	4		
Grey	MediaPipe	.489 ±.124	.497	6	1,035.252	<.001
	OpenPose	.481 ±.169	.517	5		
	PCT	.615 ±.134	.64	4		
	RTMpose	.666 ±.136	.703	2		
	**Sapiens**	**.666 ±.137**	**.704**	**1**		
	VitPose	.63 ±.164	.677	3		

*Results of the Kruskal–Wallis test

**Fig. 8:**
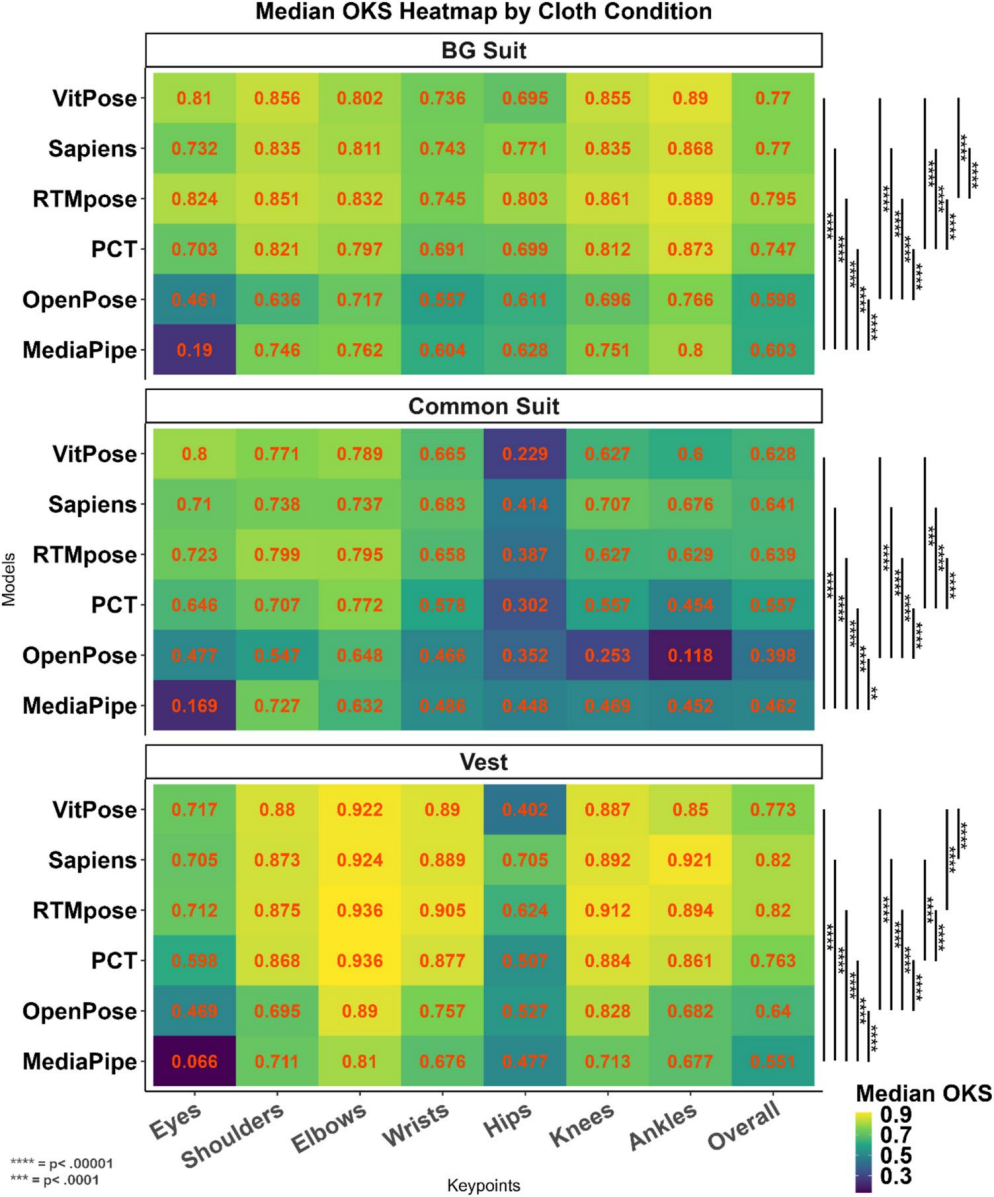
Heat maps showing the accuracy of different methods for the babygrow (BG) (top), common onesie (middle), and vest (bottom) clothes and keypoints. Most of the models showed better performance in the vest, which had the highest contrast with the background, was not loose, showed the baby’s body correctly, and did not cover the baby’s hands and legs. Pairwise comparison between different models on overall OKS was conducted using the Wilcoxon median-rank test

Table 8 demonstrates that there was a significant difference among models (*p* <.001) in different conditions. Similar to the previous conditions, RTMpose and Sapiens demonstrated the best performance in all three conditions, and MediaPipe and OpenPose had the lowest performance (Fig. 8).

**Table 8:** The effect of clothes condition on the accuracy (OKS) of different methods

Clothes condition	Method	Mean (*SD*)	Median	Rank (median)	H	p*
Babygrow	MediaPipe	.569 ±.156	.602	4	4,096.817	<.001
	OpenPose	.564 ±.184	.598	5		
	PCT	.715 ±.137	.746	3		
	**RTMpose**	**.786 ±.082**	**.795**	**1**		
	Sapiens	.742 ±.131	.77	2		
	VitPose	.76 ±.084	.77	2		
Common onesie	MediaPipe	.455 ±.11	.462	5	628.301	<.001
	OpenPose	.424 ±.17	.397	6		
	PCT	.562 ±.13	.556	4		
	RTMpose	.617 ±.137	.638	2		
	**Sapiens**	**.616 ±.135**	**.641**	**1**		
	VitPose	.582 ±.175	.628	3		
Vest	MediaPipe	.528 ±.126	.55	5	762.845	<.001
	OpenPose	.63 ±.119	.639	4		
	PCT	.748 ±.087	.763	3		
	**RTMpose**	**.802 ±.08**	**.82**	**1**		
	**Sapiens**	**.807 ±.072**	**.82**	**1**		
	VitPose	.762 ±.077	.773	2		

*Results of the Kruskal–Wallis test

#### Tests of lighting conditions

**Fig. 9:**
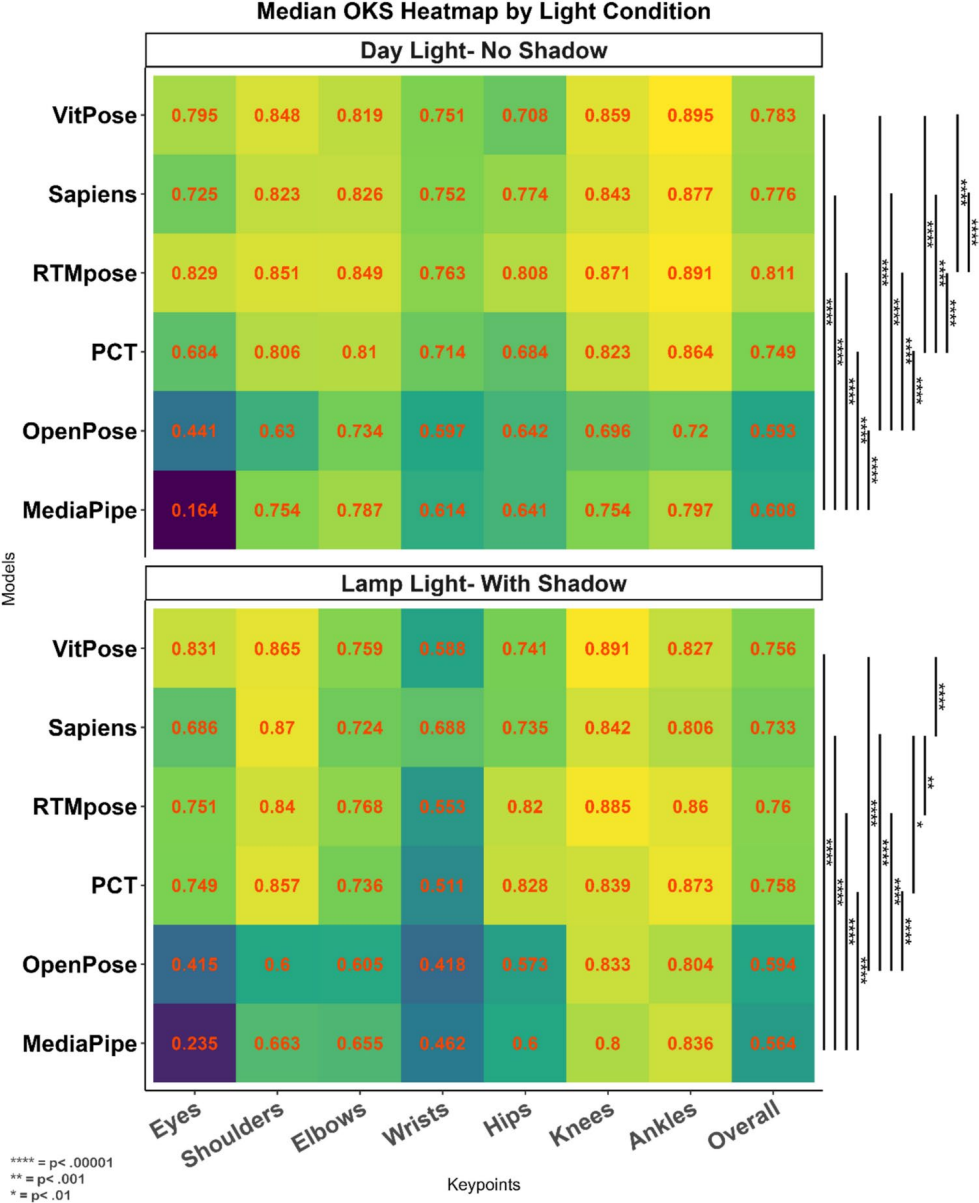
Heat maps showing the accuracy (OKS) of the methods for lighting conditions and keypoints in lamp light with shadow (top) and daylight with no shadow (bottom). All models showed better performance in the daylight/no shadow condition. Pairwise comparison between different models on overall OKS was conducted using the Wilcoxon median-rank test

The results of the Kruskal–Wallis test (Table 9) showed a significant difference between different models (*p* <.001) in each condition, and all models demonstrated better performance in daylight with no shadow. In general, for both conditions, the RTMpose achieved the best performance among methods. The heat map provided in Fig. 9 shows the detailed performance of different methods.

**Table 9:** The effect of light conditions on the accuracy (OKS) of different models

Light condition	Method	Mean (*SD*)	Median	Rank (median)	*H*	p*
Daylight, no shadow	MediaPipe	.567 ±.167	.607	5	3,124.312	<.001
	OpenPose	.558 ±.197	.593	6		
	PCT	.709 ±.149	.748	4		
	**RTMpose**	**.794 ±.083**	**.81**	**1**		
	Sapiens	.741 ±.142	.776	3		
	VitPose	.765 ±.089	.782	2		
Lamp light with shadow	MediaPipe	.55 ±.106	.564	6	763.477	<.001
	OpenPose	.56 ±.143	.593	5		
	PCT	.744 ±.073	.757	2		
	**RTMpose**	**.746 ±.079**	**.759**	**1**		
	Sapiens	.731 ±.056	.733	4		
	VitPose	.755 ±.055	.756	3		

*Results of the Kruskal–Wallis test

#### Tests of recording angle

**Fig. 10:**
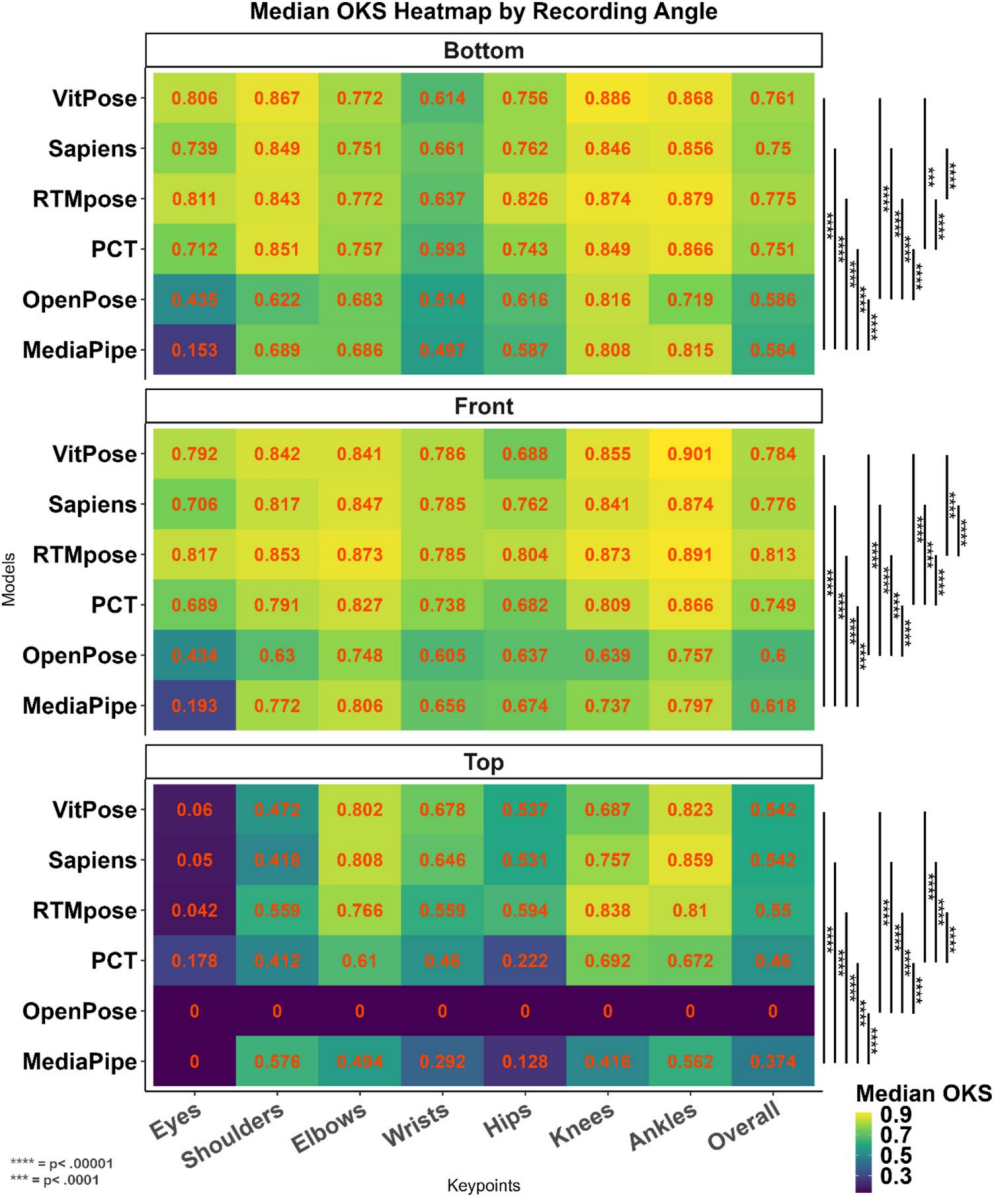
Heat maps showing the accuracy of different methods for the top (top), front (middle), and bottom (bottom) recording views and keypoints. Pairwise comparison between different models on overall OKS was conducted using the Wilcoxon median-rank test

The final factor that we considered with respect to the performance of the methods was the recording angle. As shown in Table 10, a significant difference was found among models (*p* <.001) in different recording angles, resulting in differences in accuracy levels among models, with the front view proving to be the best recording angle for all methods. When the top view was used for the recording, VitPose was the best method and OpenPose was the least accurate method, with close to zero successful detections. For the front and bottom recording views, similar to most of the previous conditions, the RTMpose method was the best. Figure 10 shows the accuracy of the various methods in the detection of different keypoints with the different recording views.

**Table 10:** The effect of recording angle on the accuracy (OKS) of different methods

Recording angle	Method	Mean (*SD*)	Median	Rank (median)	*H*	p*
Bottom	MediaPipe	.541 ±.124	.564	6	1,651.436	<.001
	OpenPose	.57 ±.118	.585	5		
	PCT	.731 ±.09	.751	3		
	**RTMpose**	**.764 ±.092**	**.774**	**1**		
	Sapiens	.739 ±.098	.75	4		
	VitPose	.754 ±.086	.761	2		
Front	MediaPipe	.578 ±.176	.618	5	2,243.481	<.001
	OpenPose	.551 ±.223	.6	6		
	PCT	.705 ±.162	.749	4		
	**RTMpose**	**.799 ±.076**	**.812**	**1**		
	Sapiens	.74 ±.149	.775	3		
	VitPose	.769 ±.084	.784	2		
Top	MediaPipe	.326 ±.176	.373	5	343.953	<.001
	OpenPose	.004 ±.019	0	6		
	PCT	.439 ±.115	.46	4		
	RTMpose	.546 ±.111	.55	2		
	Sapiens	.538 ±.064	.541	3		
	**VitPose**	**.536 ±.168**	**.678**	**1**		

*Results of the Kruskal–Wallis test

Further descriptive results regarding the performance distribution among the models in the different conditions and keypoints are provided in the supplementary file S3.

## Discussion and implications

The results of this study showed that, overall, RTMpose was the most accurate method for pose estimation in newborns, followed by VitPose and Sapiens, PCT, MediaPipe, and OpenPose. However, the accuracy of these models was lower than the AP calculated for the same method in COCO dataset tests by other authors (Lan et al., 2023) on older participants, with 80.9 reported by the VitPose developers (Xu et al., 2022) and 78.8 by the RTMpose developers (Jiang et al., 2023). Most models were able to detect a higher rate of AP50, which shows that they were able to detect a human body in the frames; however, when the AP increased to 90, the detection rate decreased significantly, indicating that these models were not sufficiently accurate to estimate the landmark locations on the frames. This may be related to the difference in body size between the infants and the trained models. This study highlights the limitations of these methods in pose detection for infants and the need to consider the factors associated with infant videos when developing HPE models in the future.

Furthermore, we showed how different video conditions affect the model performance. Almost all models performed better in the older babies, which may be because all these models were trained based on the annotated images of older samples. In the early weeks after birth when a baby’s body scale is very different from an adult’s (Villar et al., 2014), these models may struggle to correctly estimate the proportions of the baby’s body skeleton. Another important factor that affects pose estimation accuracy shown by this study is the variation in backgrounds and clothings. Analysing videos of babies in natural settings with less control makes pose detection challenging for HPE methods. Some combinations of background and clothing make discrimination between the body and the background difficult. During the home video recording sessions, infants are laid in the supine position in order to capture the whole-body movement repertoire. As such, the contrast between their clothes and background is an important factor for capturing the fine detail of each limb and allowing the discrimination between the baby and the floor. In particular, the discrimination between the infants’ clothes and the background helps shape the correct bounding box or heat map for later skeleton representation by models. For instance, in the colourful background (see supplementary file S5), the contrast between the baby’s clothes and background was low, resulting in less accurate detections for the trunk keypoints (shoulders and hips). This is in comparison to the arm and leg keypoints (elbows, wrists, knees, and ankles), which were uncovered and contrasted well with the background. The combination of colourful background and clothing also made the instance detection in the frames more challenging for many methods and increased their redundancy rate (see supplementary file S5). On the other hand, in the common onesie condition, the models were less accurate, as the baby was on a grey background and the onesie may have been loose, thus failing to show the baby’s body shape correctly. This made the shape heat map, produced by models, difficult to recognise. Furthermore, in the common onesie, the baby’s feet were covered, which may have contributed to a confusing body segmentation and made the detection of the ankles more difficult for most of the models. This study also found that lighting affects the performance of the HPE methods. Again, since newborn babies are placed in the supine position in the videos, their body creates a shadow which confuses detection for the HPE methods. We showed that all models performed better in brighter light with no shadow, mainly because in some conditions the body’s shadow was interpreted as another person by the model or as the limb in the frame (see supplementary file S4). Abbasi et al. (2023) showed the negative effect of high-contrast shadow on the body part labelling function of their model. Also, our findings of recording angle are consistent with their finding indicating less accurate detections in the tilted camera recordings, compared with the front camera view, which achieves the most accurate HPE for almost all models.

Looking at the processing speed results, MediaPipe proved to be the fastest method and Sapiens the slowest. Although we expected RTMpose to show a faster processing speed (real-time speed), it was not as fast as MediaPipe. Considering different ratios of speed and accuracy as the efficiency factor, when the ratio was 70:30 (accuracy/speed), MediaPipe and RTMpose emerged as the superior methods, with VitPose, PCT, Sapiens, and OpenPose following them. When we increased the speed weight in the efficiency calculation to 50 and 70, the speed of MediaPipe compensated for its limitations in accurate detection. This highlights the computation cost of HPEs, especially in studies with high sampling rates and a large number of videos. Using MediaPipe may save time, which is a possible reason that many researchers are motivated to use this method in their studies, regardless of its lower accuracy. We also showed several conditions that can significantly affect the performance of different models which were not mentioned in the literature, and we recommend that future studies consider these factors in their design in order to improve the quality of data collection. We next discuss the performance of each model, including practical insights, limitations, and tips from our experience with each method.

### MediaPipe

While MediaPipe (Lugaresi et al., 2019) had the lowest accuracy, its most noteworthy strengths were its speed, ease of use, and broad compatibility. It is designed to function on various devices, including mobile devices and PCs, across different operating systems, which makes it a desirable method for researchers and users. MediaPipe provides three pre-trained models (lite, full, and heavy); we selected the heavy model for this analysis to enable a fair comparison with other models. Face detection is central to pose estimation in MediaPipe (Lugaresi et al., 2019). Since newborn babies lack control over their heads, their face is often turned to the side, and MediaPipe therefore struggles to detect the faces correctly. As a result, the body direction and pose estimation will not be accurate, as evidenced by the current investigation. We believe this limitation contributed to its weaker performance. This is an important factor for future users to consider during data collection.

### OpenPose

OpenPose (Cao et al., 2021) is one of the most widely recognised pose estimation methods in developmental psychology, likely due to its accessibility and its comprehensive tutorial resources on GitHub, making it user-friendly for researchers with limited programming skills. The demo version does not require complex installation (aside from Python) and can run on both CPU and GPU versions; however, the GPU version is significantly faster and requires a compatible PC. OpenPose offers two pre-trained models (COCO and MPII); we used the COCO model in this study. Future researchers may wish to investigate the MPII model to assess its accuracy and speed on infants. It is important to note that in frames where the baby was recorded upside-down, OpenPose struggled with pose detection, and this should be considered in future research.

### PCT, RTMpose, Sapiens, and VitPose

These four methods share certain commonalities and distinctions. Primarily, they were developed using the MMPose (Chen et al., 2020), MMDet (Chen & Ran, 2019), and MMCV (MMCV Contributors, 2018) toolboxes and libraries. In the top-down method used in this study, they employ MMDetection to detect humans within frames and draw bounding boxes around each detected individual, followed by MMPose to identify probable marker locations. RTMpose is also compatible with RTMdet for drawing bounding boxes; however, we found that it is less stable and makes redundant detections in frames in comparison to MMDetection (please see supplementary file S6 for further information and examples). The difference between these methods lies in their calculations of keypoint probabilities based on their respective trained models and calculations. Some methods, such as PCT, demonstrate the ability to estimate keypoints under challenging conditions. It should be noted that all of these methods have different pre-trained models, and we only considered the models recommended by the previous studies or by the method developers. For example, Sapiens (Khirodkar et al., 2024) has another heavier model which was trained by 3 billion interactions, but due to very slow processing speed, we did not consider it in our analysis. VitPose has another pre-trained model called VitPose Plus, which we tried to use but were unsuccessful due to several errors related to the incompatibility of Python packages with our GPU driver, and there were no solutions in their GitHub repository to solve these issues. Future studies may want to consider these models if they have a faster and more efficient GPU on their machine.

### The battle of compatibility and redundant detections

Despite their superior performance in pose estimation, the state-of-the-art methods (PCT, RTMpose, Sapiens, and VitPose) presented several challenges related to installation and data post-processing. Firstly, installation instructions were not straightforward, requiring a certain level of expertise. These methods have multiple dependencies, all of which require correct installation, including CUDA, PyTorch, MMPose, MMCV, and MMDetection. CUDA versions are dependent on the GPU driver version installed on the machine. On Windows PCs, the latest GPU drivers install the latest CUDA version, although most of these HPE methods are compatible with older CUDA versions. Downgrading CUDA on Windows is complex, albeit simpler on Linux, making Linux or Windows Subsystem for Linux (WSL) a preferable option for using these methods. This requirement, however, is not stated in the official instructions for these models, which may deter researchers interested in using these methods and encourage them to use the less accurate methods (MediaPipe and OpenPose). In the supplementary file S7, we provide detailed installation instructions for these dependencies.

Another issue, noted in this study and a recent study by Gama et al. (2025), is redundant detections/instances where the algorithm detects more poses in a frame than are actually present. State-of-the-art methods have been designed for pose estimation in crowd conditions, meaning that they can detect multiple people in each frame, which may cause false detections. Such redundancies can compromise the reliability of detections if proper post-processing is not applied. We encountered several cases in which these methods detected multiple people in a frame, particularly when colourful backgrounds or the infant’s shadow were present. Although PCT performed well against colourful backgrounds, it also had the highest rate of redundant detections (see supplementary files S4 and S5). These issues complicate detection when the method alternates between real and redundant detections, potentially resulting in false detections. We also showed that MediaPipe and OpenPose had no redundant detections, although they had missing frames. Fortunately, most of these methods have the option to set up the maximum number of instances/persons in each frame. To deal with the redundant detections, we recommend setting a reasonable number of instances for each method and a careful post-processing protocol involving visual validation of detections. Fortunately, these methods all offer the capability to visualise their detections as either pictures or video.

### Limitations

This study had several limitations which should be addressed. Although we wanted to test these models on a diverse range of ethnicities, our participants were mostly from Caucasian ethnic groups, and researchers should be cautious about generalising these findings to other ethnic groups. With the rapid technological advancements and artificial intelligence (AI), it was difficult to consider all the available state-of-the-art algorithms in this study; future studies might test the function of the other models. Also, it was not possible to use these algorithms with all available pre-trained models, such as MPII or YOLO, which might be interesting for future researchers.

## Conclusion

With rapid technological advancements, new methods and models continually emerge, enhancing automation and accessibility for researchers. Human pose estimation methods exemplify these technologies. In general, state-of-the-art methods can accurately estimate poses of newborns in well-controlled settings; however, our study demonstrated that there is a need for further development of HPE models tailored for naturalistic, home scenarios with less controlled environments. Until these advancements are realised, we recommend that researchers conducting infant studies implement sufficient controls during recording to facilitate later post-processing of data by increasing the contrast between babies’ bodies and background, recording their video with a standardised recording angle between 45° and 90°, and preferably in a onesie which is not loose or a vest which shows the baby’s full body with limited shadows.

## Supplementary Information

Below is the link to the electronic supplementary material.

Supplementary file1 (DOCX 8580 KB)

Supplementary file2 (PDF 5272 KB)

## Data Availability

All data reported in this research are openly available from the University of Sussex Figshare at [https://figshare.com/s/d2f4c12f77a5734ab553].

## Code availability

Codes will be available from the University of Sussex Figshare at [https://figshare.com/s/d2f4c12f77a5734ab553].
