# Supplementary material for: Automatic pose estimation in newborn infants: Lessons from the Baby Grow study
Source: Behav Res Methods. 2026 Mar 9;58(3):82. doi: 10.3758/s13428-026-02943-z (PMC12971853; doi:10.3758/s13428-026-02943-z)
Supplement: Supplementary file 1 — Supplementary file1 (DOCX 8580 KB) [file 13428_2026_2943_MOESM1_ESM.docx]

**S1**

**Models description**

- **MediaPipe Pose**: Developed by Google, MediaPipe detects 33 body keypoints in 3D coordinates. Unlike most HPE models which provide the location of each keypoint in 2D (x and y coordinates), the MediaPipe provides the 3D coordination for each keypoint (x, y and z) although Z-axis precision is limited. MediaPipe offers three pre-trained models (Lite, Full and Heavy) and is compatible with both CPU and GPU (Graphic Processing Unite or Graphic Card). The MediaPipe Pose solution has been designed to run a fast pose detection with the application for mobile devices ([https://github.com/google-ai-edge/MediaPipe?tab=readme-ov-file](https://github.com/google-ai-edge/mediapipe?tab=readme-ov-file)).
- **OpenPose**: OpenPose is the first real-time multi-person system for detecting human bodies and joints. It supports full-body mode (with a maximum of 135 keypoints) and pose detection mode (17 keypoints for the COCO dataset and 25 for the MPII dataset). The OpenPose could be run on both CPU and GPU. The demo version of OpenPose has been designed as an Exe file to run easily on the PC without any previous Python package installations and is compatible with all operating systems (Windows, Linux and Mac) ([https://cmu-perceptual-computing-lab.github.io/OpenPose/web/html/doc/index.html](https://cmu-perceptual-computing-lab.github.io/openpose/web/html/doc/index.html)).
- **PCT**: Human Pose as Compositional Tokens (PCT) is a structural representation exploring joint dependencies. PCT has been designed to detect human pose for challenging occlusions based on joint dependencies. PCT uses a classifier to predict a category of M tokens from images to estimate each keypoint’s position. PCT uses the MS COCO dataset and requires specific NVIDIA GPUs (<https://sites.google.com/view/pctpose>).
- **RTMpose**: Real Time Models for Pose estimation (RTMpose) is an optimised project from MMPOSE. RTMpose employs top-down approach to estimate the pose for each individual in the frame in a real time speed. It has received an excellent performance on public benchmarks, yet its application for infant studies is not clear. It works both on CPU and GPU,with an AP score= 78.8 for its largest model for COCO dataset (Jiang et al., 2023). (https://github.com/open-mmlab/mmpose/tree/main/projects/rtmpose).
- **Sapiens**: Recently released by META®, Sapiens models perform four human-centric vision tasks, including 2D pose estimation. Sapiens’ efficiency increases with model size, though the 2-billion-parameter model was too heavy for our PC. It has been trained by the MS COCO dataset and needs a GPU for pose detection. The Demo was designed to detect pose on the images rather than videos. (<https://github.com/facebookresearch/sapiens>).
- **VitPose**: VitPose uses vision transformers as backbones with a lightweight decoder for pose estimation, available in Basic, Large, and Huge models. It is trained on the MS COCO dataset and runs on both CPU and GPU depending on dependencies (<https://github.com/ViTAE-Transformer/ViTPose>).

**S2**

**ICC results between GT and independent coders**

| **GT and Coder 1** | | | | | **GT and Coder 2** | | | | |
| --- | --- | --- | --- | --- | --- | --- | --- | --- | --- |
| **Limb** | **ICC Type** | **ICC Value** | **p-value** | **95% CI** | **Limb** | **ICC Type** | **ICC Value** | **p-value** | **95% CI** |
| right_eye_x | ICC1 | 0.99933 | 0 | [1. 1.] | right_eye_x | ICC1 | 0.99955 | 0 | [1. 1.] |
| right_eye_y | ICC1 | 0.99994 | 0 | [1. 1.] | right_eye_y | ICC1 | 0.99977 | 0 | [1. 1.] |
| right_shoulder_x | ICC1 | 0.99553 | 0 | [0.99 1. ] | right_shoulder_x | ICC1 | 0.99691 | 0 | [1. 1.] |
| right_shoulder_y | ICC1 | 0.9969 | 0 | [1. 1.] | right_shoulder_y | ICC1 | 0.99866 | 0 | [1. 1.] |
| right_elbow_x | ICC1 | 0.99912 | 0 | [1. 1.] | right_elbow_x | ICC1 | 0.9989 | 0 | [1. 1.] |
| right_elbow_y | ICC1 | 0.99472 | 0 | [0.99 1. ] | right_elbow_y | ICC1 | 0.99765 | 0 | [1. 1.] |
| right_wrist_x | ICC1 | 0.99916 | 0 | [1. 1.] | right_wrist_x | ICC1 | 0.99886 | 0 | [1. 1.] |
| right_wrist_y | ICC1 | 0.99108 | 0 | [0.99 0.99] | right_wrist_y | ICC1 | 0.99799 | 0 | [1. 1.] |
| right_hip_x | ICC1 | 0.99652 | 0 | [1. 1.] | right_hip_x | ICC1 | 0.99427 | 0 | [0.99 1. ] |
| right_hip_y | ICC1 | 0.9954 | 0 | [0.99 1. ] | right_hip_y | ICC1 | 0.97808 | 0 | [0.97 0.98] |
| right_knee_x | ICC1 | 0.99707 | 0 | [1. 1.] | right_ankle_x | ICC1 | 0.99931 | 0 | [1. 1.] |
| right_knee_y | ICC1 | 0.98983 | 0 | [0.99 0.99] | right_ankle_y | ICC1 | 0.99874 | 0 | [1. 1.] |
| right_ankle_x | ICC1 | 0.99671 | 0 | [1. 1.] | left_eye_x | ICC1 | 0.99908 | 0 | [1. 1.] |
| right_ankle_y | ICC1 | 0.99262 | 0 | [0.99 0.99] | left_eye_y | ICC1 | 0.99971 | 0 | [1. 1.] |
| left_eye_x | ICC1 | 0.9993 | 0 | [1. 1.] | left_shoulder_x | ICC1 | 0.99255 | 0 | [0.99 0.99] |
| left_eye_y | ICC1 | 0.9998 | 0 | [1. 1.] | left_shoulder_y | ICC1 | 0.99798 | 0 | [1. 1.] |
| left_shoulder_x | ICC1 | 0.99008 | 0 | [0.99 0.99] | left_elbow_x | ICC1 | 0.9976 | 0 | [1. 1.] |
| left_shoulder_y | ICC1 | 0.9973 | 0 | [1. 1.] | left_elbow_y | ICC1 | 0.99618 | 0 | [0.99 1. ] |
| left_elbow_x | ICC1 | 0.99689 | 0 | [1. 1.] | left_wrist_x | ICC1 | 0.99542 | 0 | [0.99 1. ] |
| left_elbow_y | ICC1 | 0.99769 | 0 | [1. 1.] | left_wrist_y | ICC1 | 0.99559 | 0 | [0.99 1. ] |
| left_wrist_x | ICC1 | 0.99709 | 0 | [1. 1.] | left_hip_x | ICC1 | 0.99628 | 0 | [0.99 1. ] |
| left_wrist_y | ICC1 | 0.99757 | 0 | [1. 1.] | left_hip_y | ICC1 | 0.99211 | 0 | [0.99 0.99] |
| left_hip_x | ICC1 | 0.99061 | 0 | [0.99 0.99] | left_knee_x | ICC1 | 0.999 | 0 | [1. 1.] |
| left_hip_y | ICC1 | 0.99642 | 0 | [0.99 1. ] | left_knee_y | ICC1 | 0.99649 | 0 | [1. 1.] |
| left_knee_x | ICC1 | 0.99872 | 0 | [1. 1.] | left_ankle_x | ICC1 | 0.99898 | 0 | [1. 1.] |
| left_knee_y | ICC1 | 0.99788 | 0 | [1. 1.] | left_ankle_y | ICC1 | 0.99905 | 0 | [1. 1.] |
| left_ankle_x | ICC1 | 0.99702 | 0 | [1. 1.] | Limb | ICC Type | ICC Value | p-value | 95% CI |
| left_ankle_y | ICC1 | 0.99806 | 0 | [1. 1.] | right_eye_x | ICC1 | 0.99955 | 0 | [1. 1.] |

**S3**

**Strip plots and statistics for the distribution of detections in different Conditions/Methods/Keypoints**

| **Overall detections** |
| --- |
| **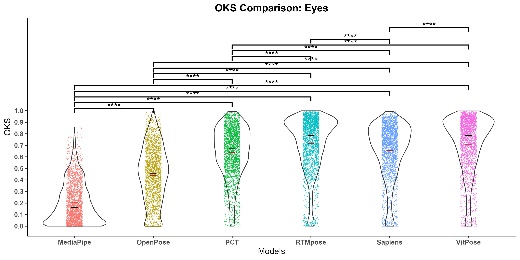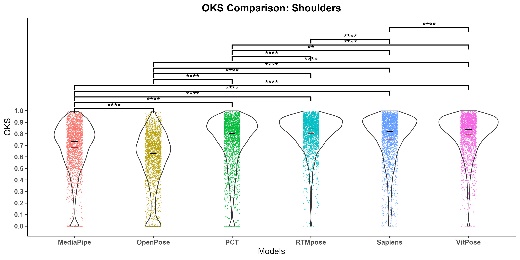** |
|  |
| **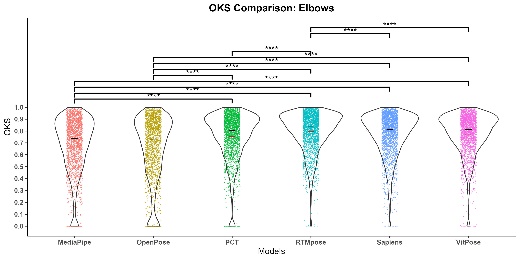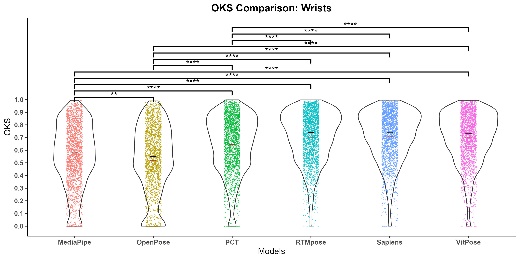** |
|  |
| **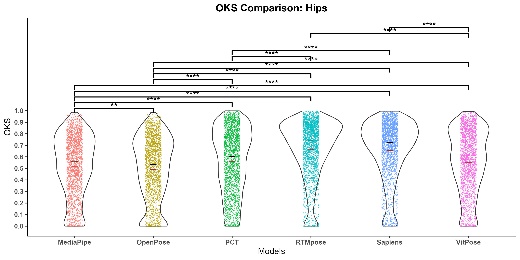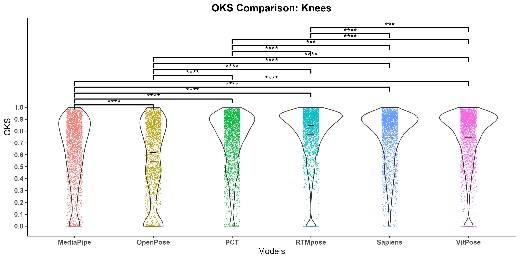** |
|  |
| **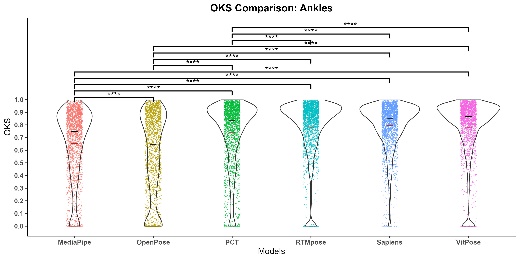** |

**Statistics results can be found at this address in the main repository:**

**Repository\Calculations\OKS\Plots\Overall_Detections\Joints**

| **Age Condition** |
| --- |
| **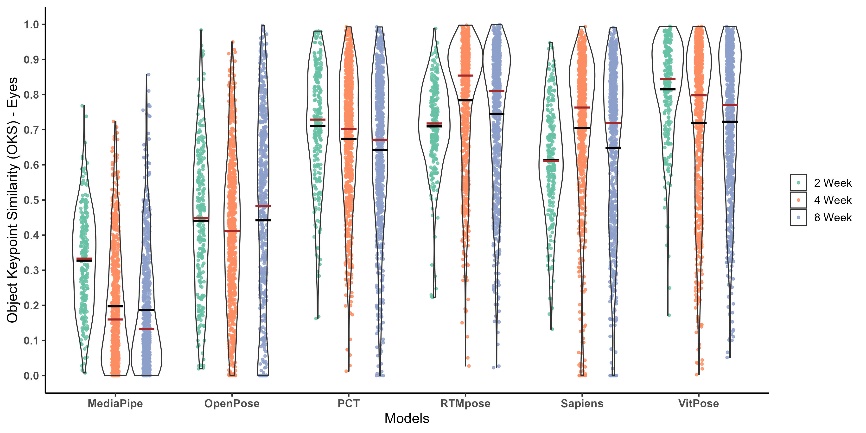** |
| **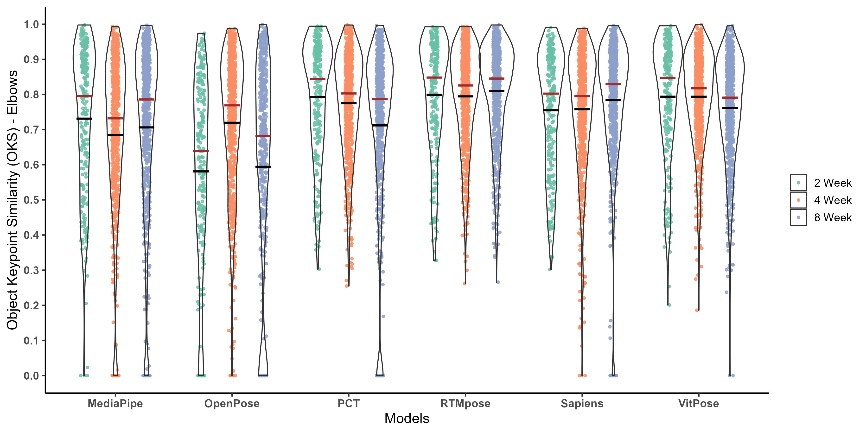** |
| **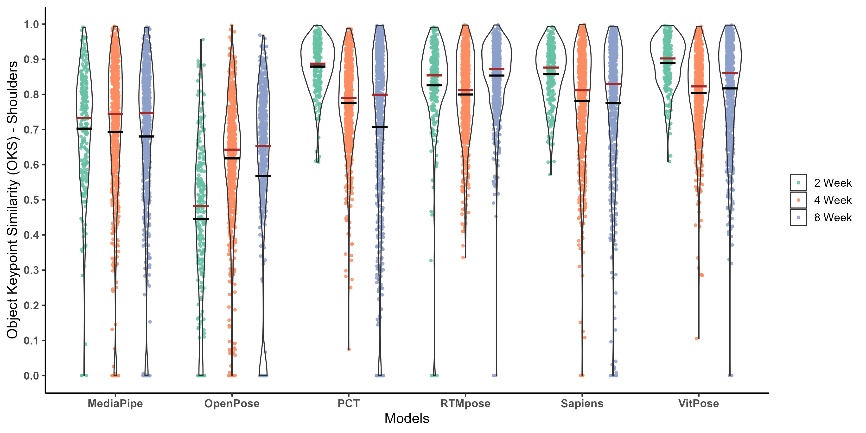** |
| **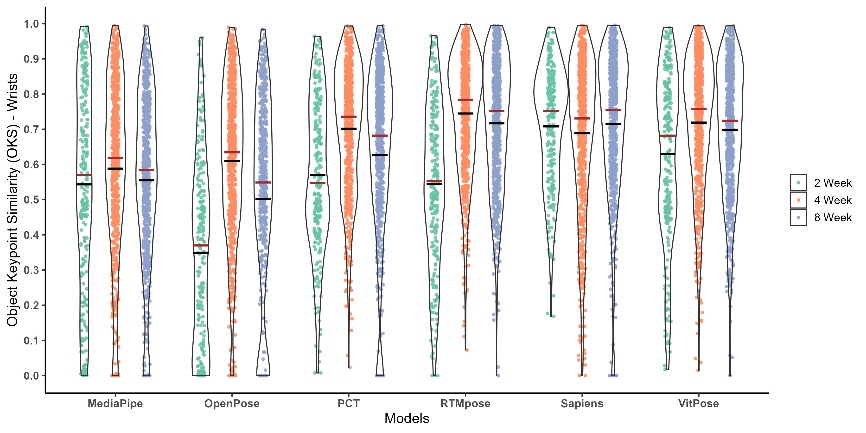** |

| **Age Condition** |
| --- |
| **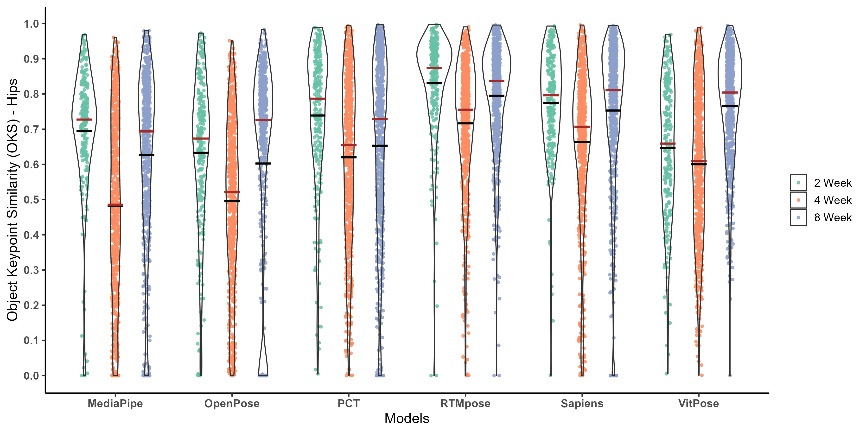** |
| **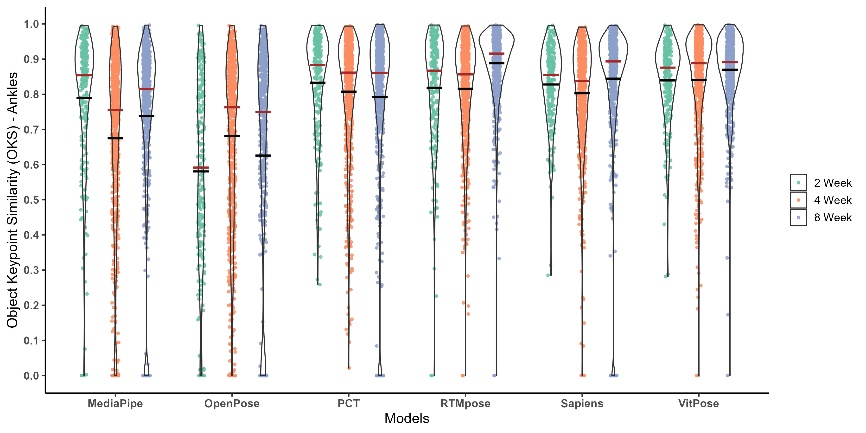** |
| **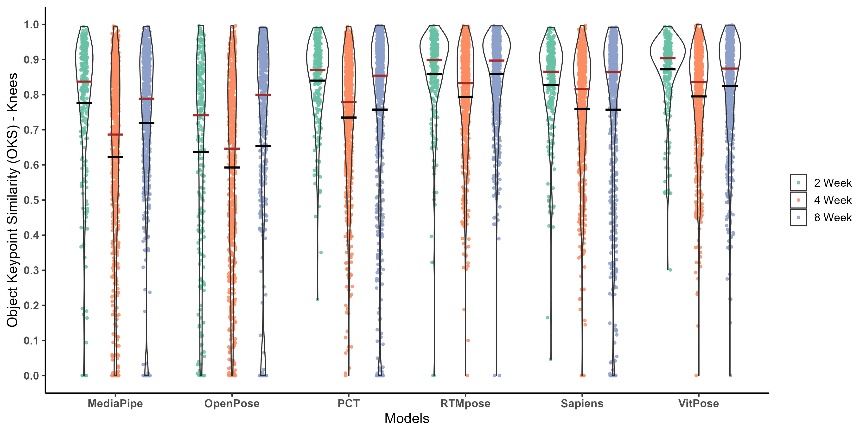** |

**Further statistical comparisons are available in our repository:**

**Repository\Calculations\OKS\Plots and stats\Age Condition**

| **Background Condition** |
| --- |
| **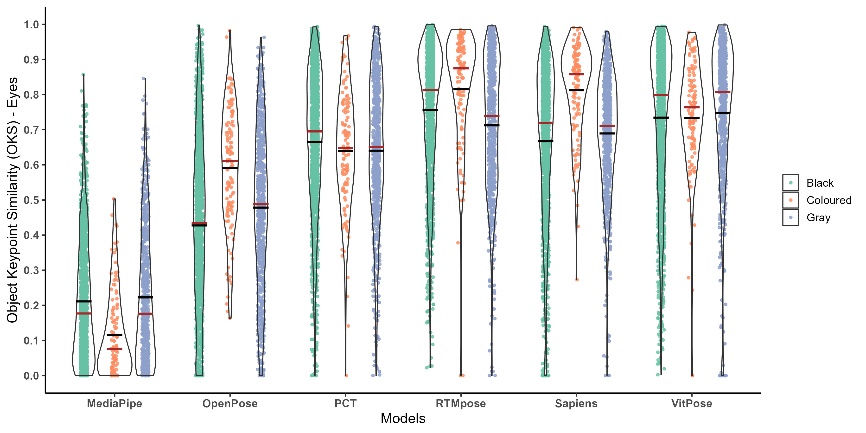** |
| **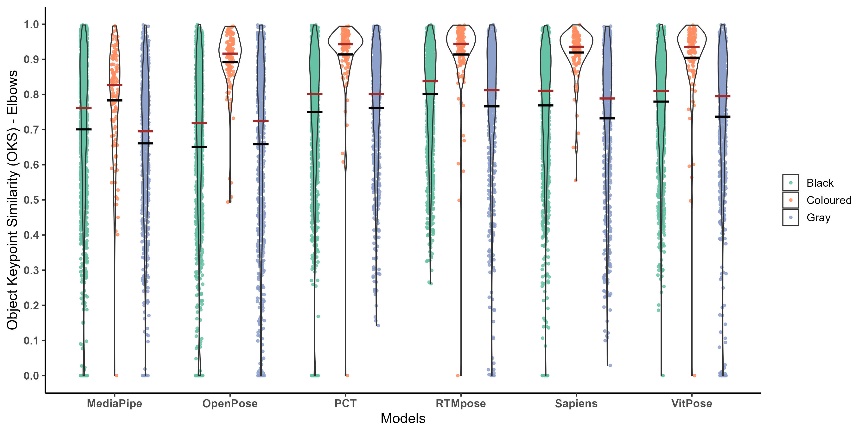** |
| **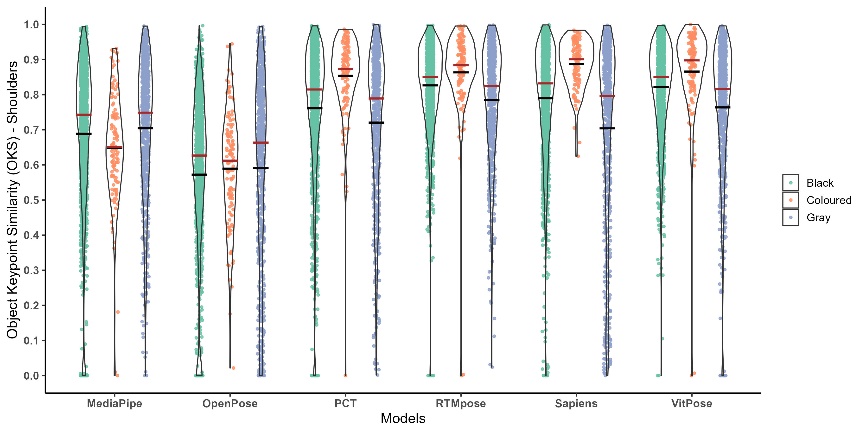** |
| **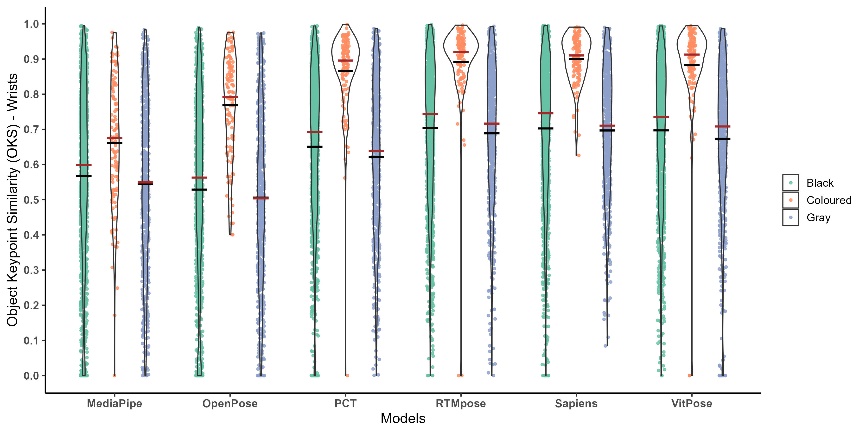** |

| **Background Condition** |
| --- |
| **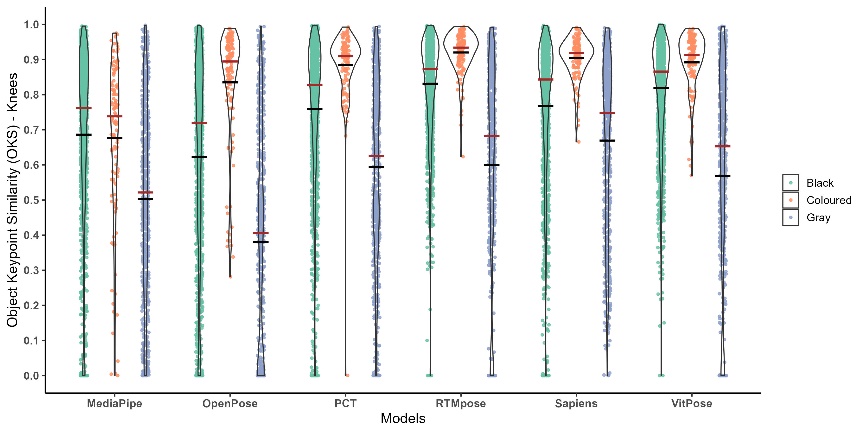** |
| **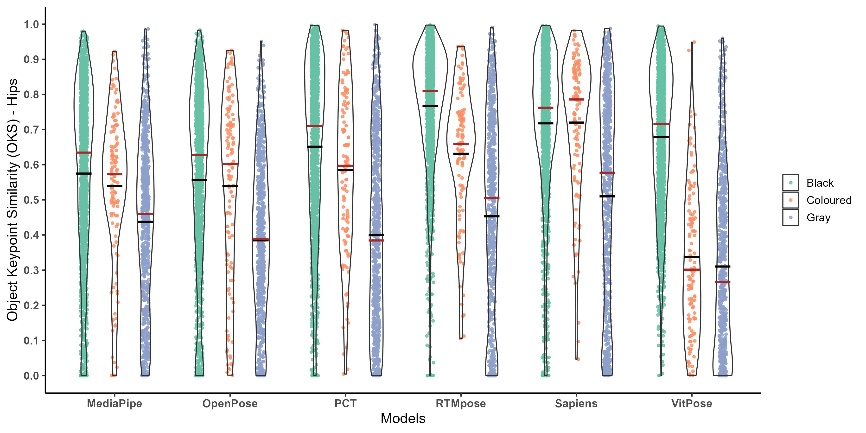** |
| **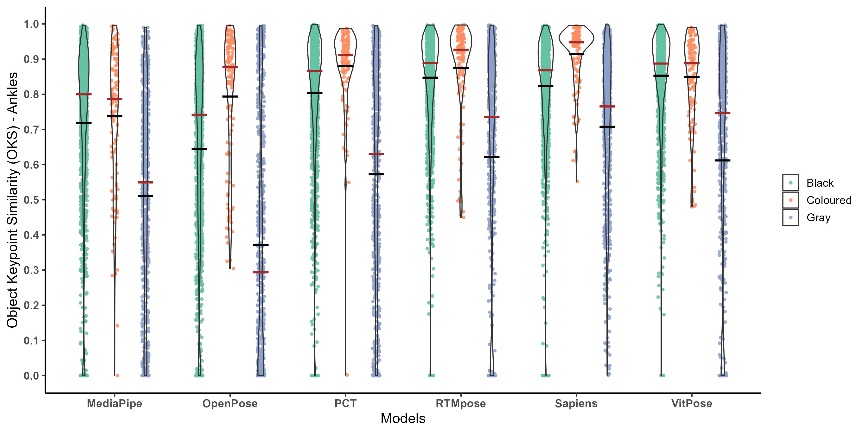** |

**Further statistical comparisons are available in our repository:**

**Repository\Calculations\OKS\Plots and stats\Background Condition**

| **Cloth Condition** |
| --- |
| **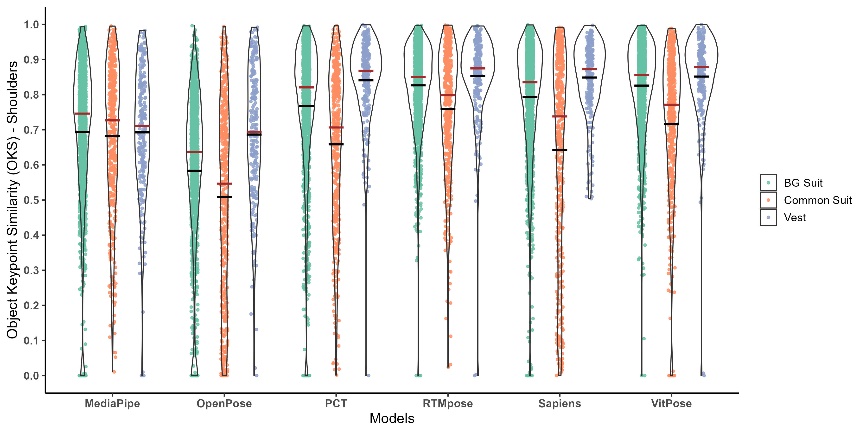** |
| **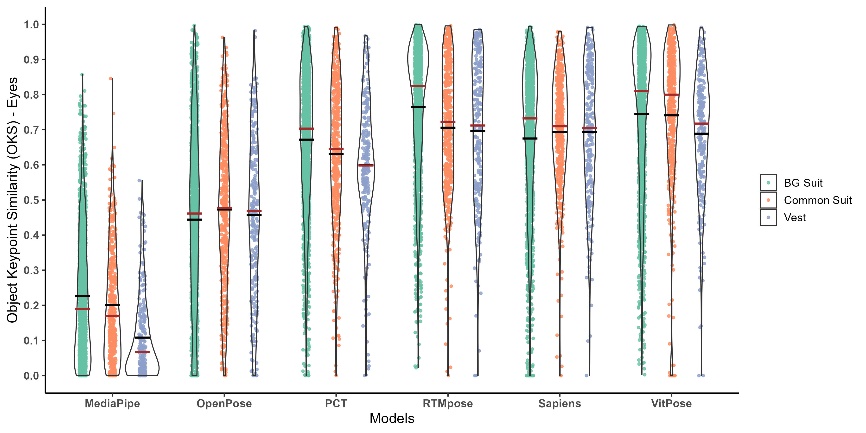** |
| **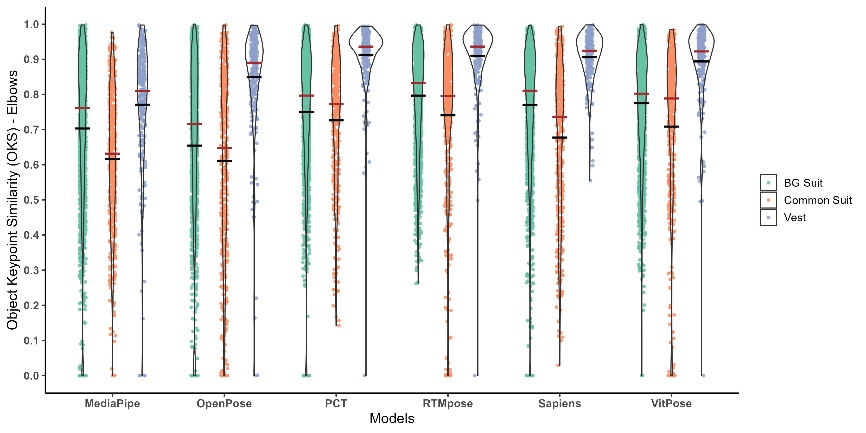** |
| **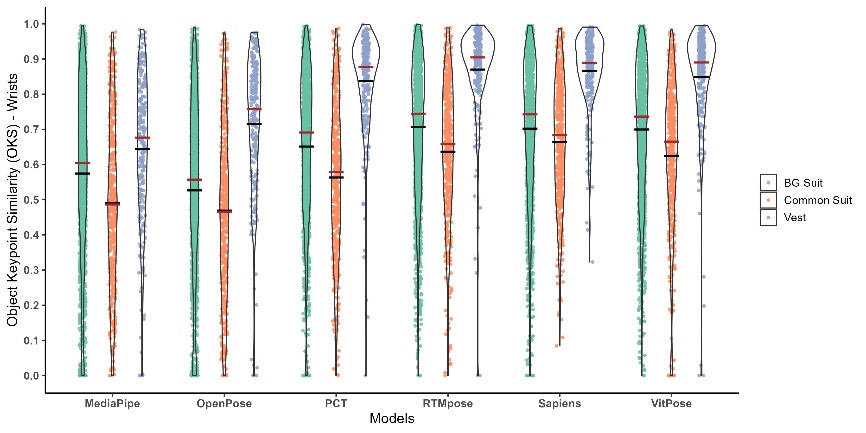** |

| **Cloth Condition** |
| --- |
| **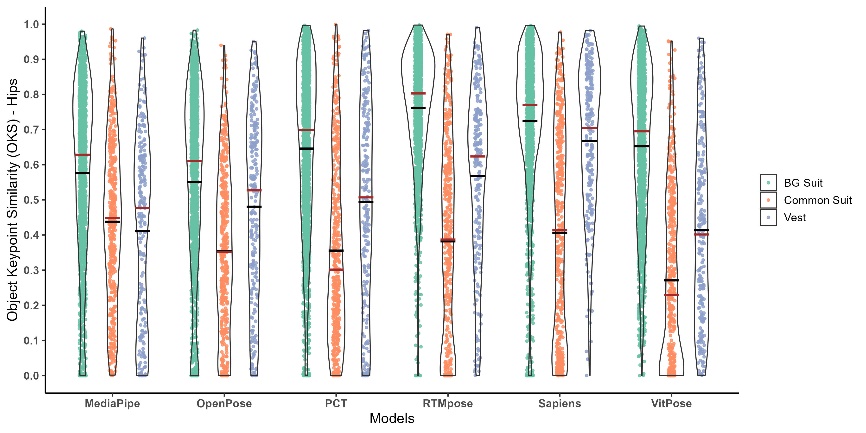** |
| **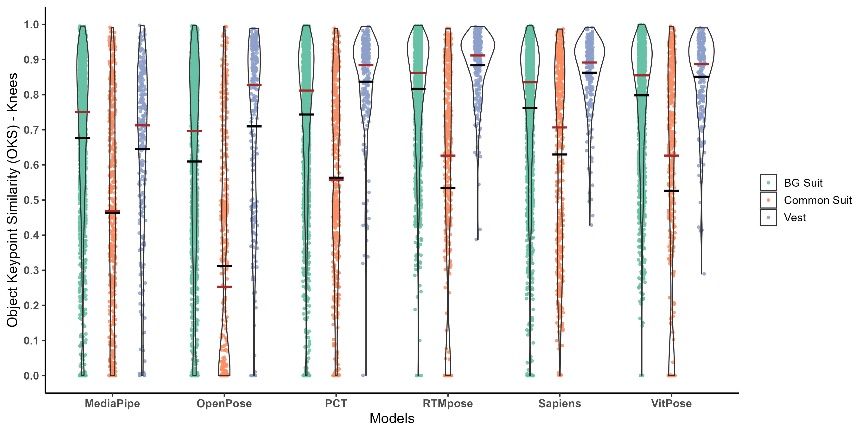** |
| **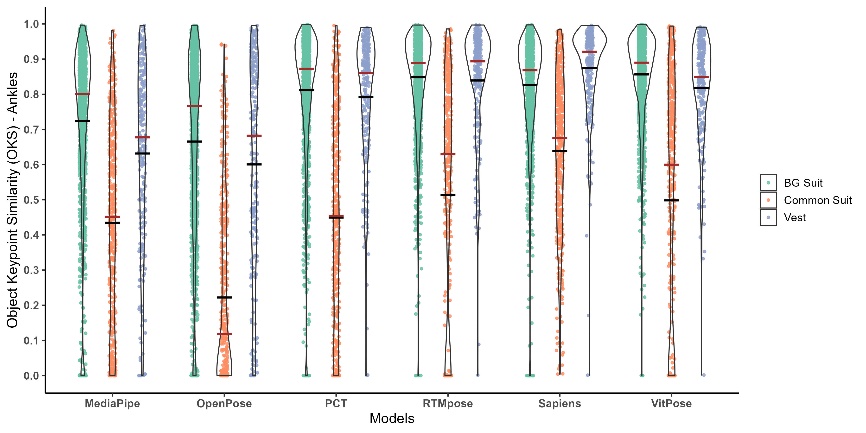** |

**Further statistical comparisons are available in our repository:**

**Repository\Calculations\OKS\Plots and stats\Cloth Condition**

| **Light Condition** |
| --- |
| **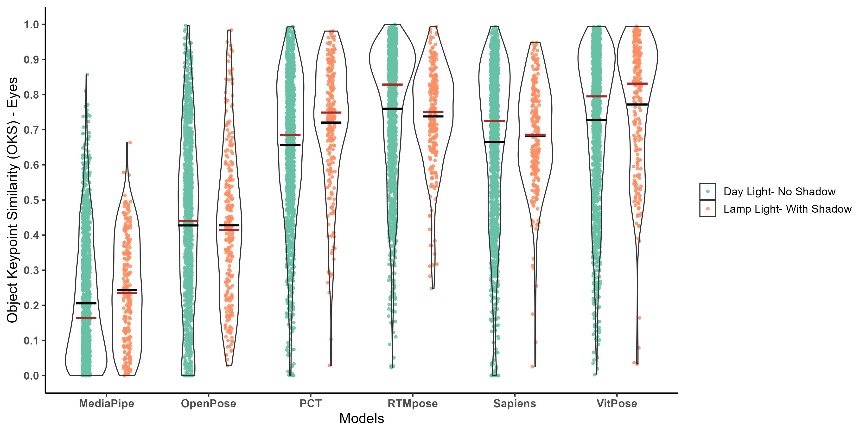** |
| **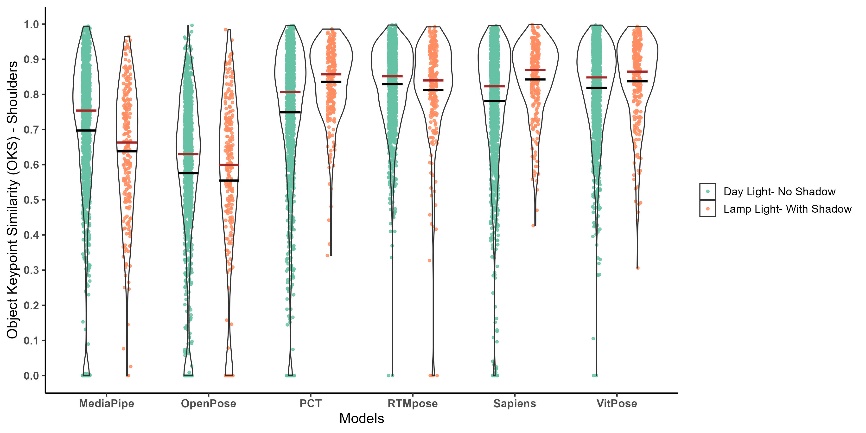** |
| **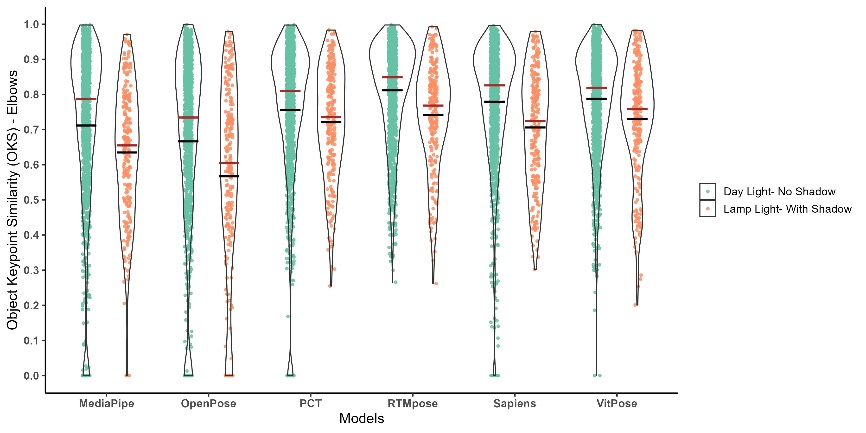** |
| 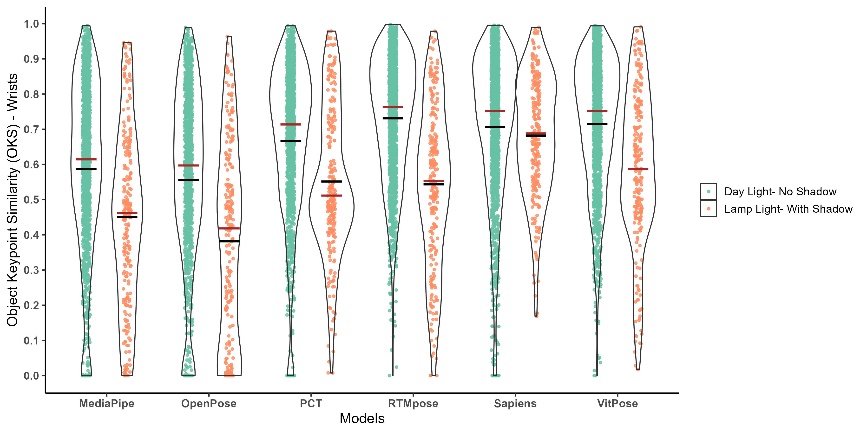 |

| **Light Condition** |
| --- |
| 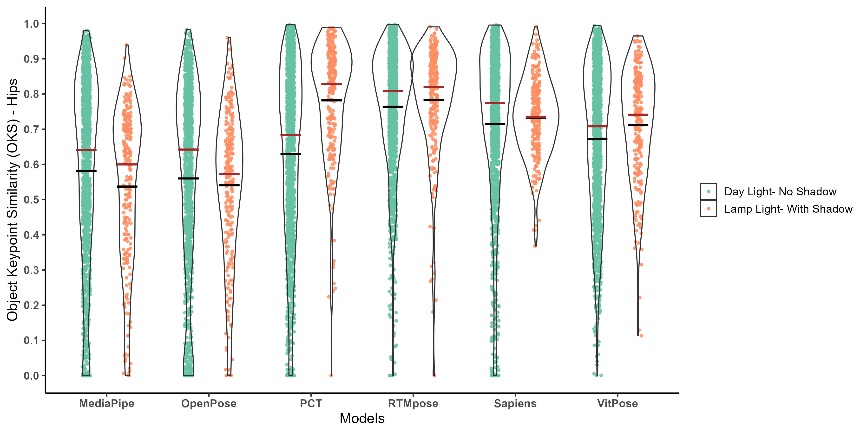 |
| 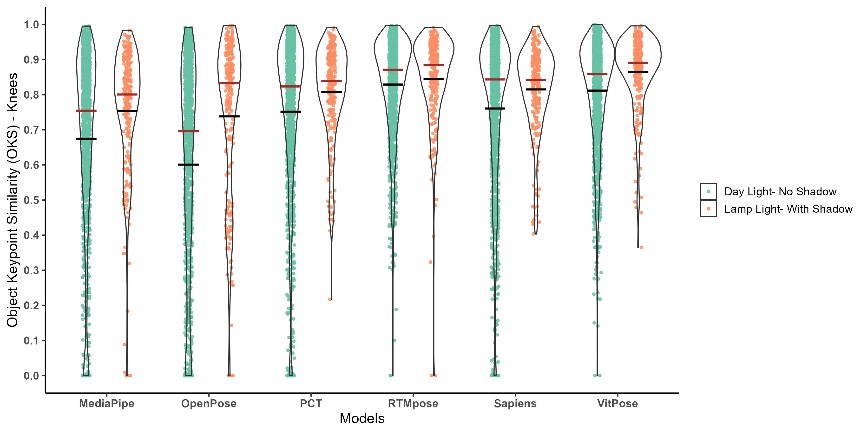 |
| 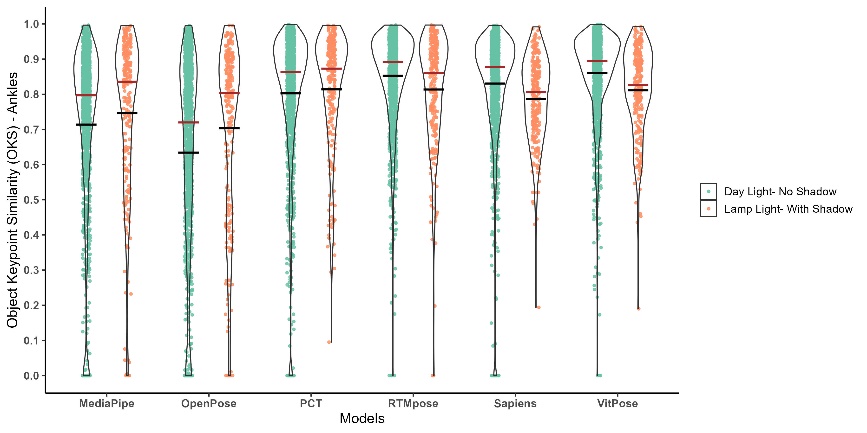 |

**Further statistical comparisons are available in our repository:**

**Repository\Calculations\OKS\Plots and stats\Light Condition**

| **Recording Angle** |
| --- |
| 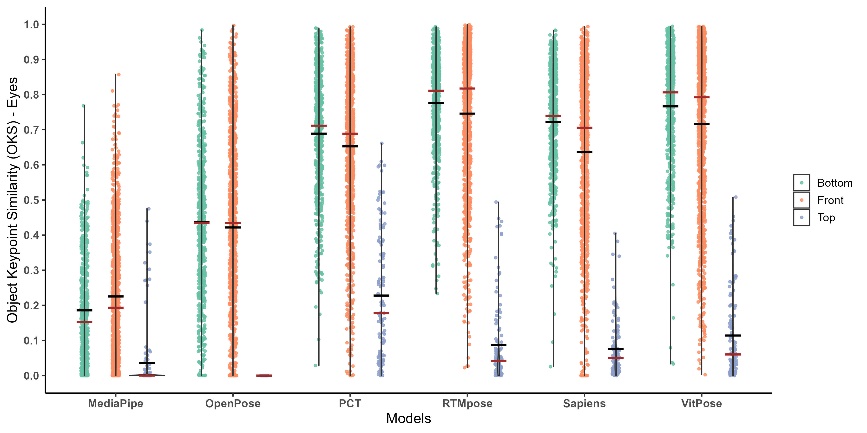 |
| 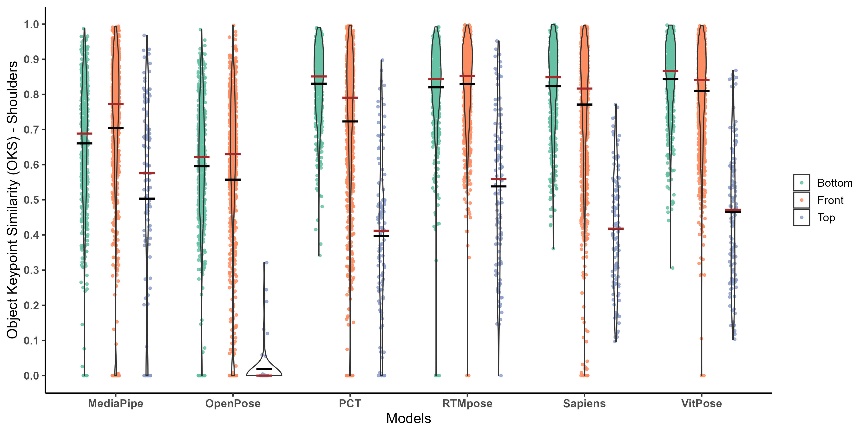 |
| 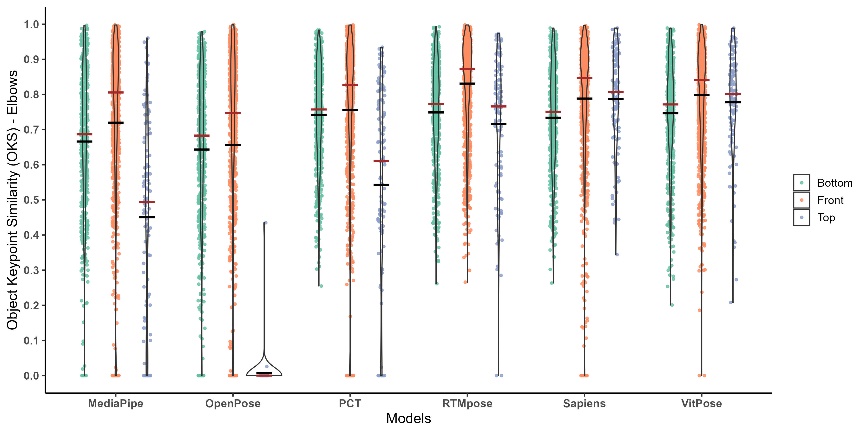 |
| 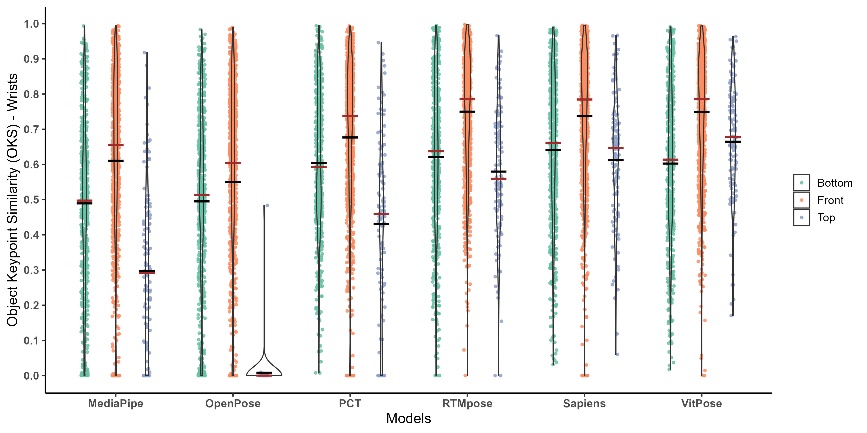 |

| **Recording Angle** |
| --- |
| 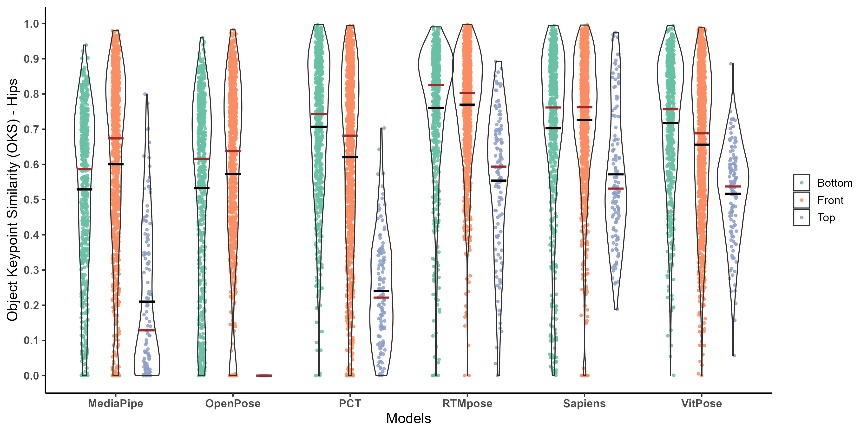 |
| 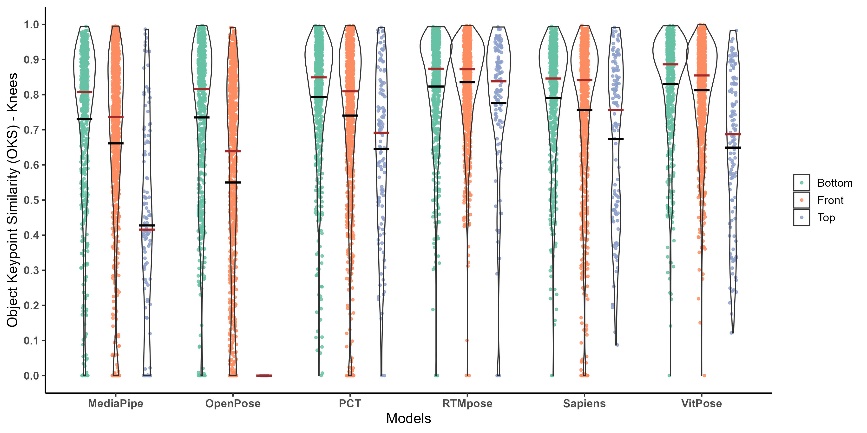 |
| 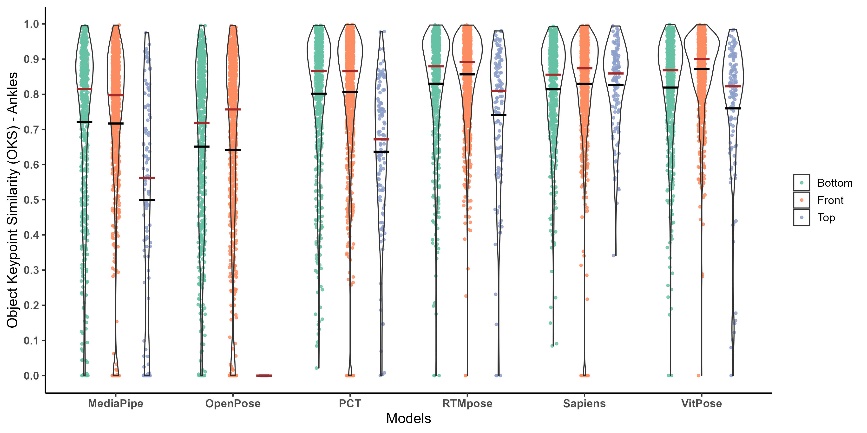 |

**Further statistical comparisons are available in our repository:**

**Repository\Calculations\OKS\Plots and stats\Recording Angle**

**S4**

**Dealing with Shadows**

| MediaPipe | OpenPose | RTMpose |
| --- | --- | --- |
| 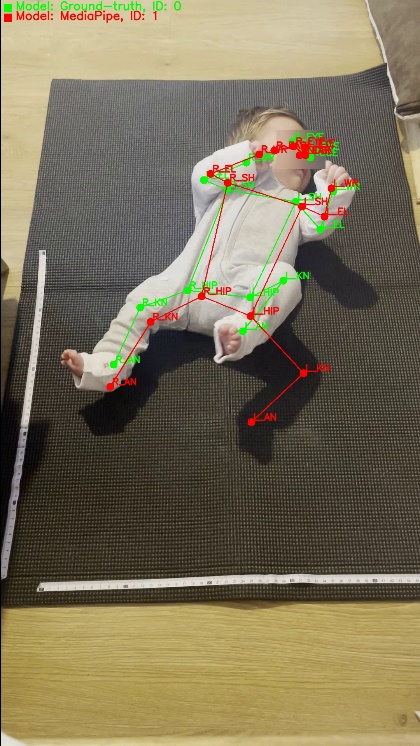 | 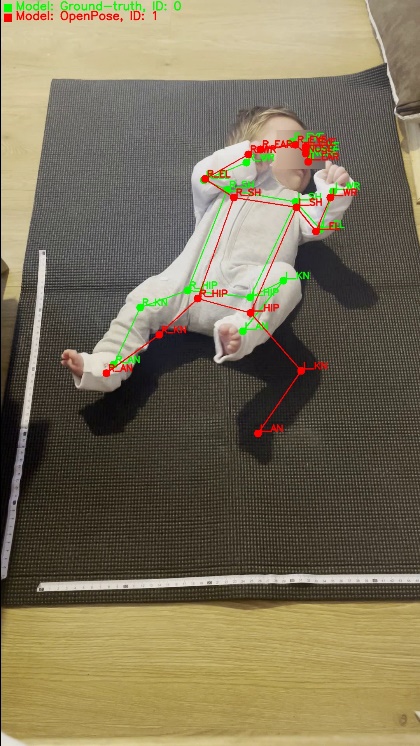 | 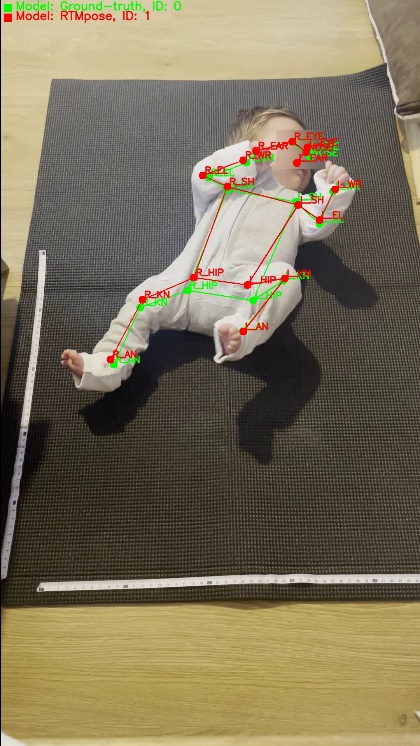 |
| PCT | Sapiens | VitPose |
| 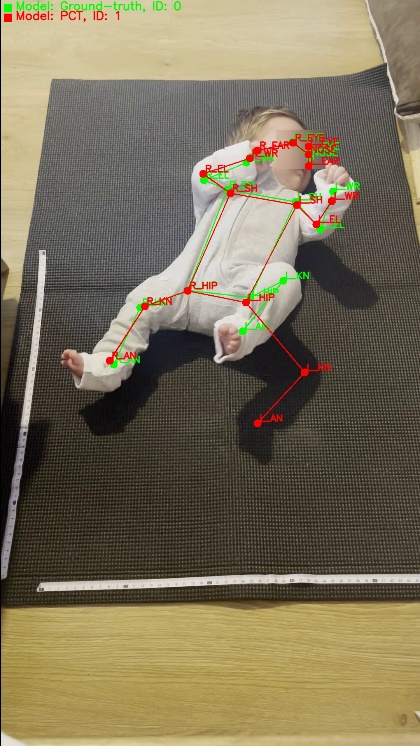 | 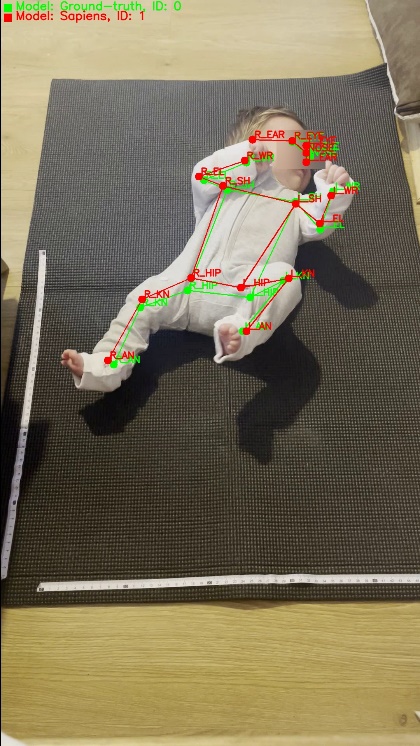 | 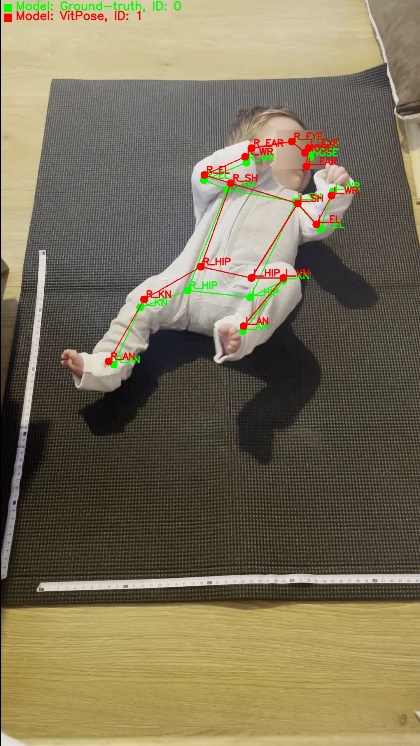 |

**These screenshots show how different models performed in a challenging Lamp Light- With Shadow condition, where the MediaPipe, OpenPose and PCT detected the baby’s shadow left leg as the baby’s leg and made a false detection for the left knee and wrist.**

**S5**

**Redundant detections**

Redundant detections are part of different methods which should be considered carefully in the HPE, especially when the discrimination of the person and background is difficult for the method or when multiple people are in the frame. These are some examples of redundant detection by PCT, Sapiens and VitPose which has detected multiple people on one frame.

| MediaPipe | OpenPose | PCT |
| --- | --- | --- |
| 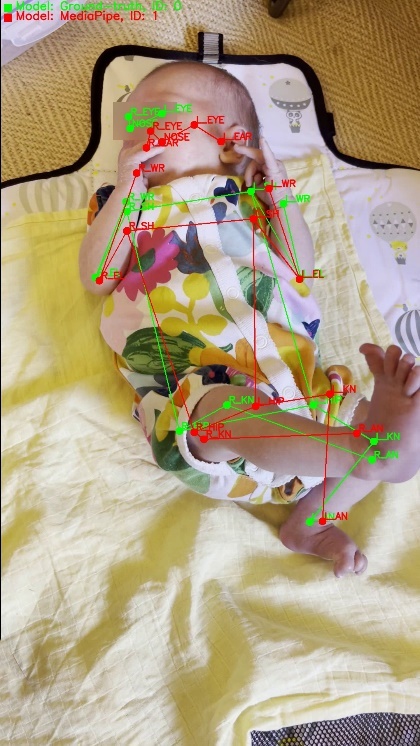 | 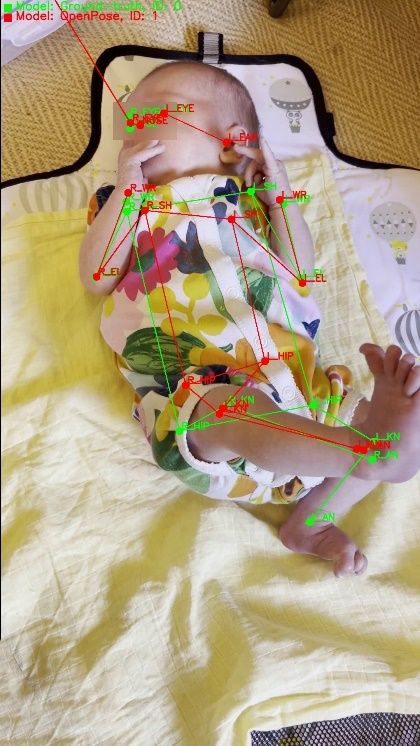 | 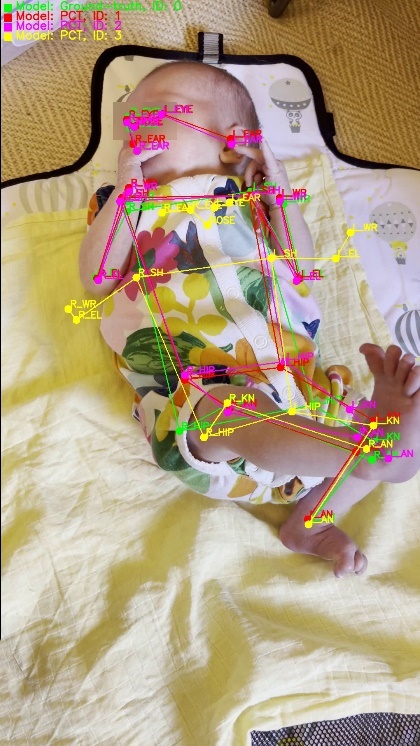 |
| RTMpose | Sapiens | VitPose |
| 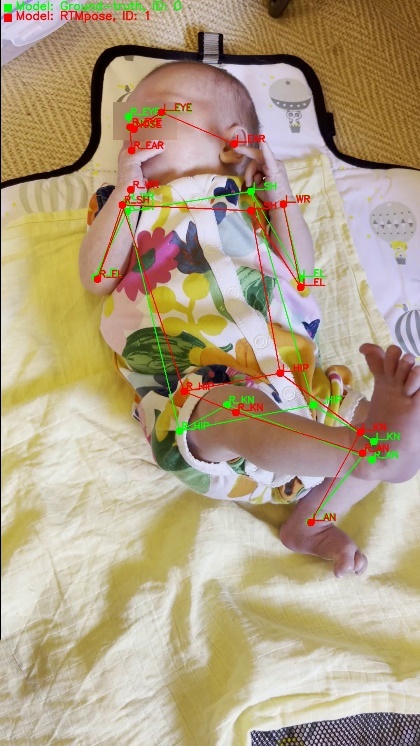 | 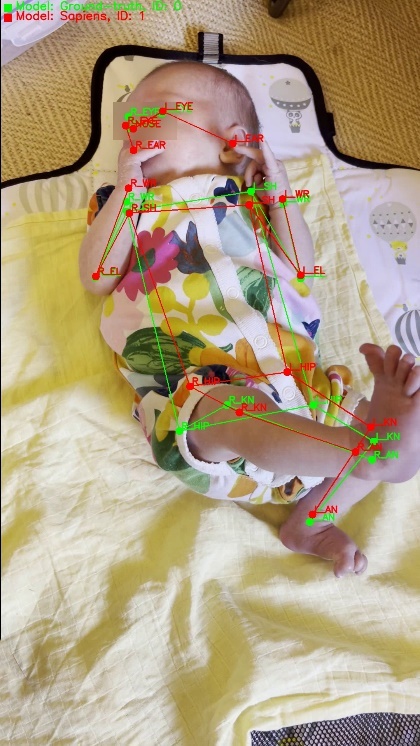 | 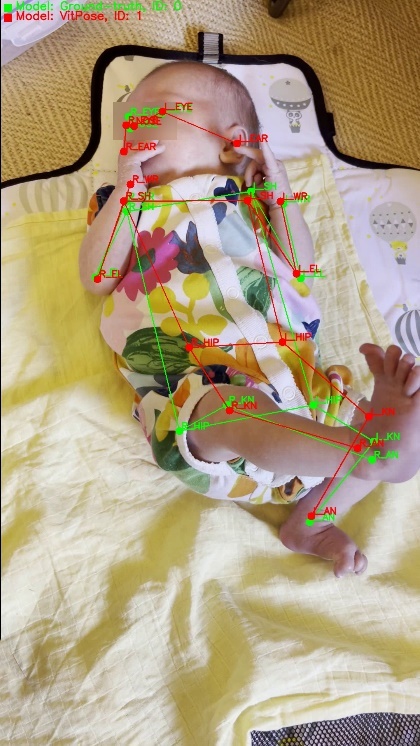 |

| MediaPipe | OpenPose | PCT |
| --- | --- | --- |
| 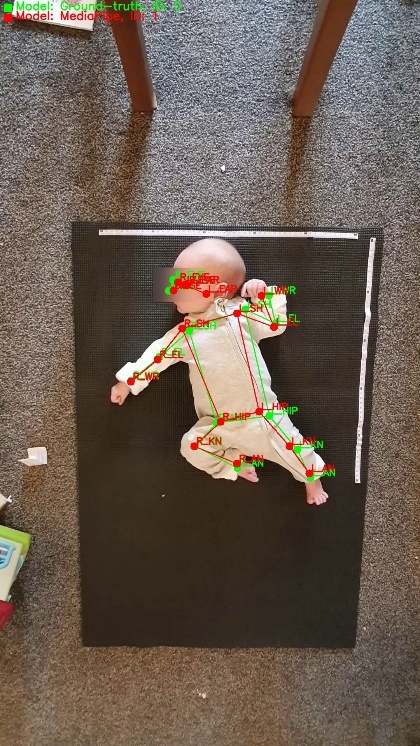 | 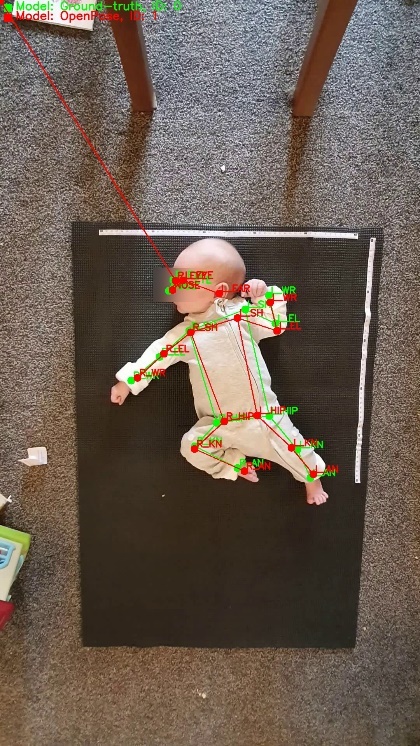 | 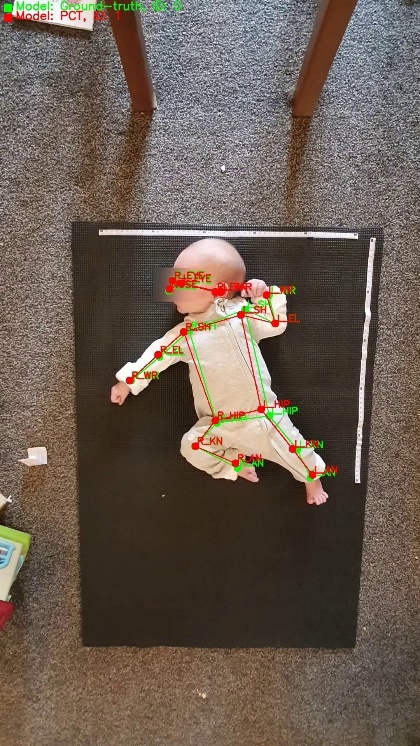 |
| RTMpose | Sapiens | VitPose |
| 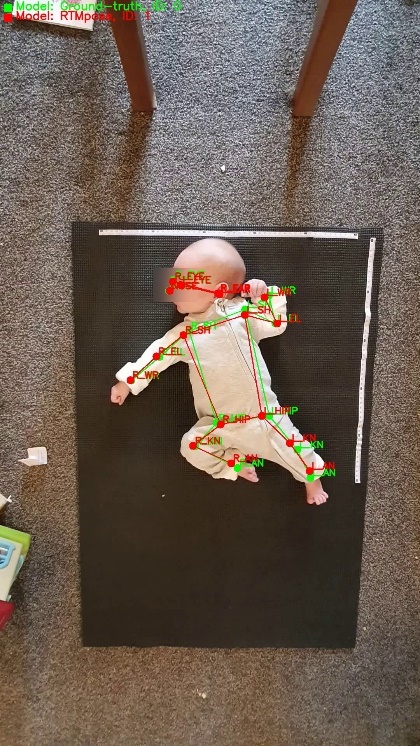 | 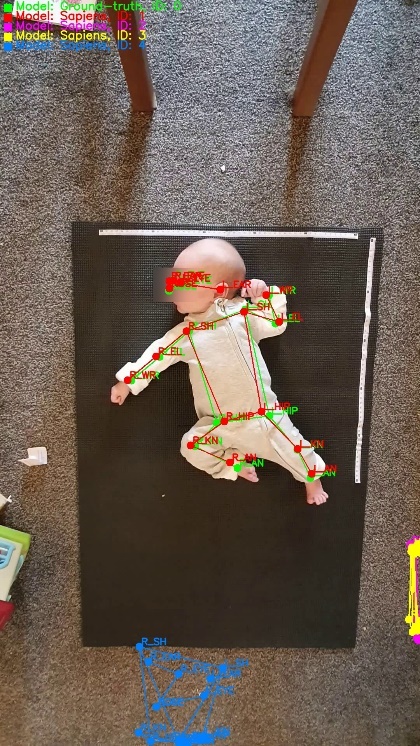 | 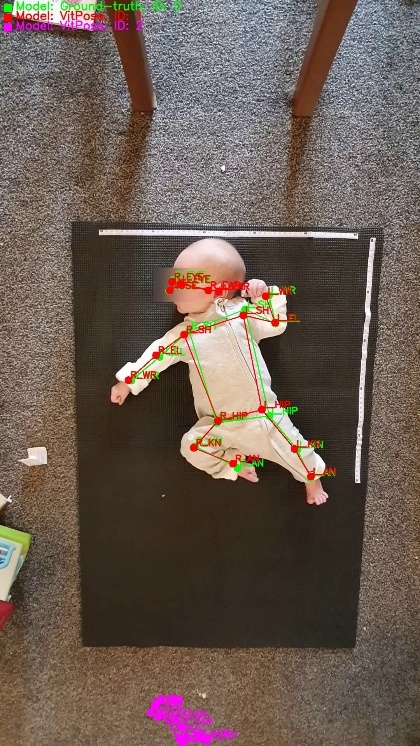 |

| MediaPipe | OpenPose |
| --- | --- |
| 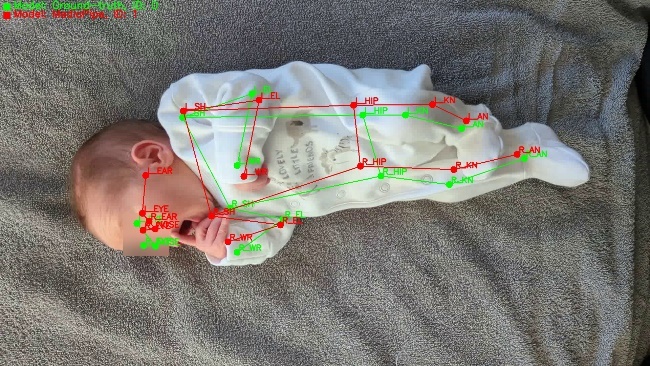 | 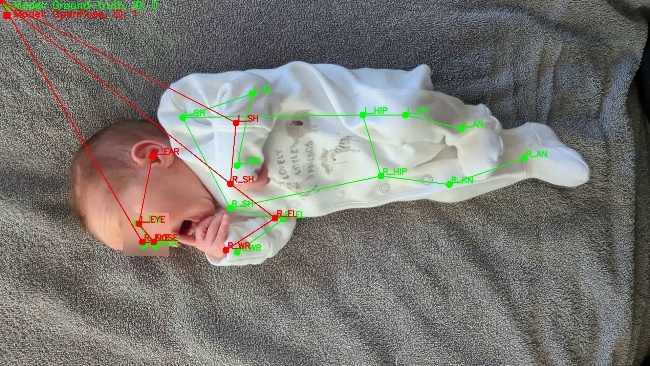 |
| PCT | RTMpose |
| 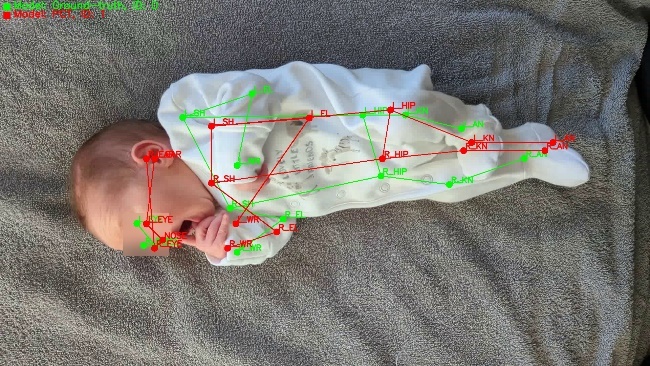 | 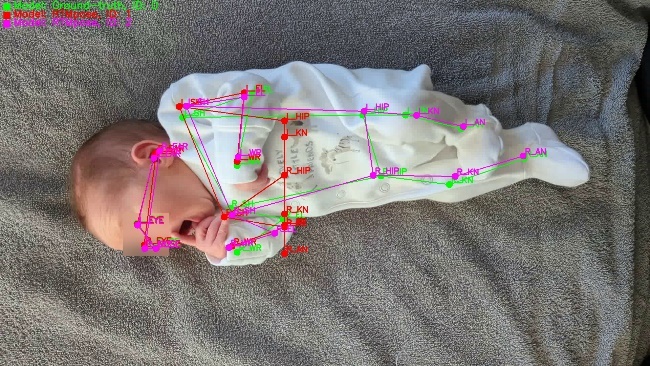 |
| Sapiens | Vitpose |
| 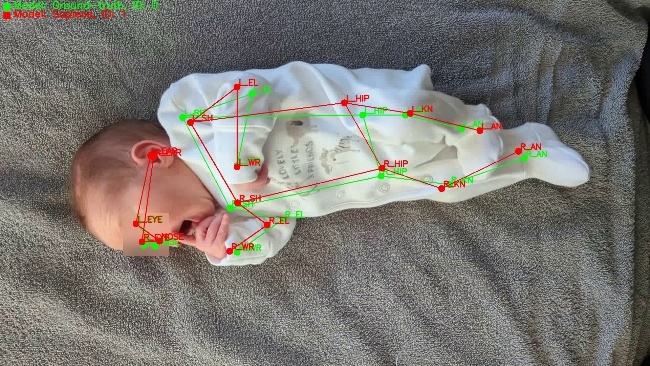 | 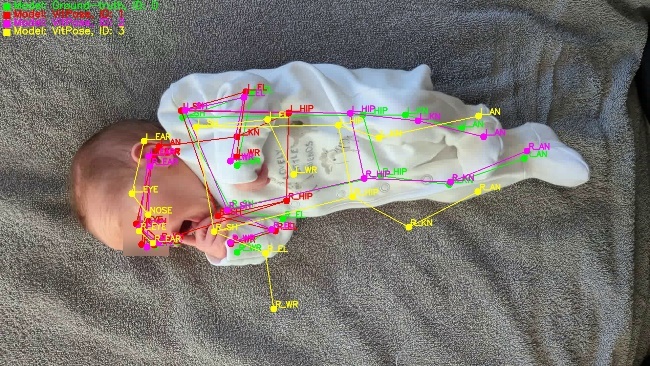 |

**S6**

**RTMpose instance detection configs**

| **RTMpose with RTMDet configs** | | |
| --- | --- | --- |
| 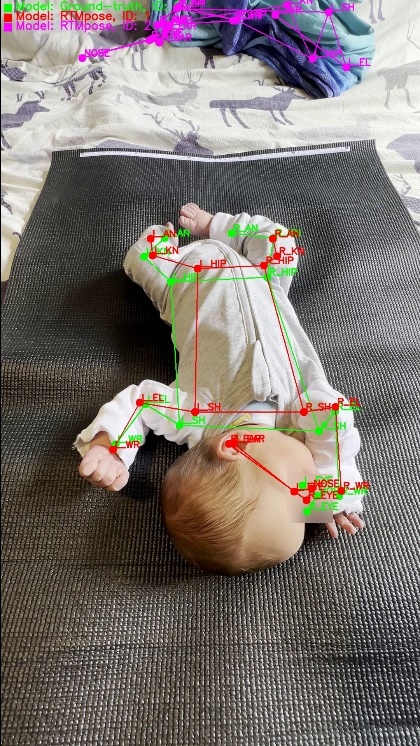 | Swapping instances with redundant detections  🡺 🡺 🡺 🡺 🡺 🡺 🡺 🡺 🡺 | 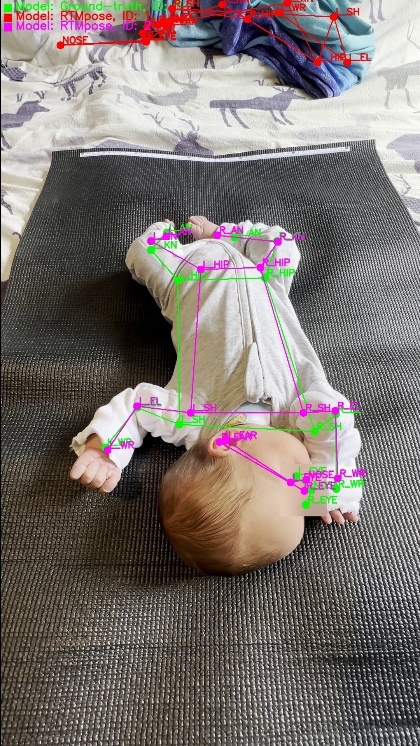 |
| **RTMpose with Mmdet configs** | | |
| 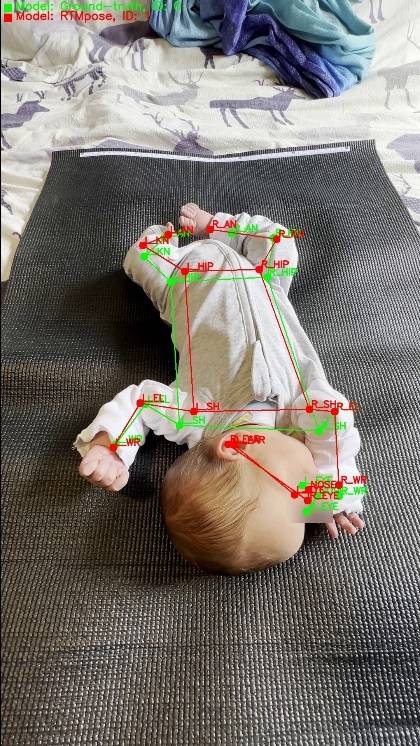 | No redundant detections in the same frames  🡺 🡺 🡺 🡺 🡺 🡺 🡺 🡺 🡺 | 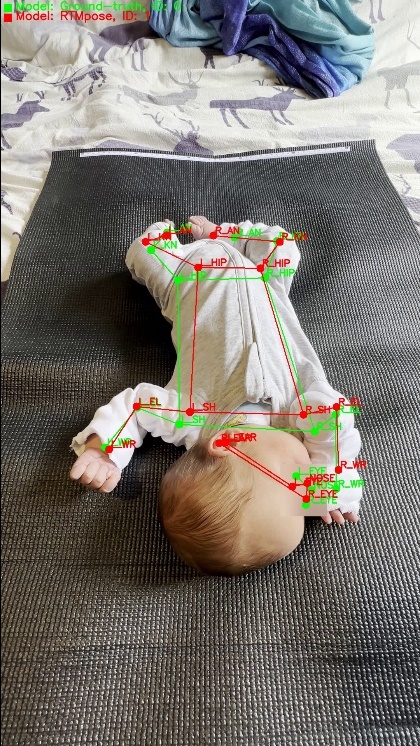 |

**S7**

**Dealing with Compatibility issues**

**RTMpose Installation For Windows users with an NVIDIA GPU machine:**

RTMpose follows the mmpose pipeline for HPE, we followed the instruction provided by RTMpose in their GitHub repository (https://github.com/open-mmlab/mmpose/tree/main/projects/rtmpose) for installation, but you will need to install mmpose gradually, from their repository in Github. (<https://mmpose.readthedocs.io/en/latest/installation.html>).

**We recommend following the below steps:**

- **Download and Install mmpose (**[**https://mmpose.readthedocs.io/en/latest/installation.html**](https://mmpose.readthedocs.io/en/latest/installation.html)**)**
- **Download RTMpose (**[**https://github.com/open-mmlab/mmpose/tree/main/projects/rtmpose**](https://github.com/open-mmlab/mmpose/tree/main/projects/rtmpose)**)**
- **Download pre-trained model for RTMpose (**<https://mmpose.readthedocs.io/en/latest/model_zoo/body_2d_keypoint.html>**)**
- **Use the sample demo files according to the instructions in this link (**<https://github.com/open-mmlab/mmpose/tree/main/projects/rtmpose>**)**

**Tips:**

RTMpose is a top-down HPE and at first it detects instances in the images and then estimates pose, we tested it with both mmdet configurations (which reported in this study) and RTMDet configs. We spotted un-stability in instance detection when RTMDet was used in redundant conditions, the model swapped instances lables which make the post processing very difficult. Therefore we recommend mmdet configs. Below is an example showing how RTMDet made redundant detection and swapped instances in comparison to mmdet.
