# Supplementary material for: Automatic pose estimation in newborn infants: Lessons from the Baby Grow study
Source: Behav Res Methods. 2026 Mar 9;58(3):82. doi: 10.3758/s13428-026-02943-z (PMC12971853; doi:10.3758/s13428-026-02943-z)
Supplement: Supplementary file 2 — Supplementary file2 (PDF 5272 KB) [file 13428_2026_2943_MOESM2_ESM.pdf]

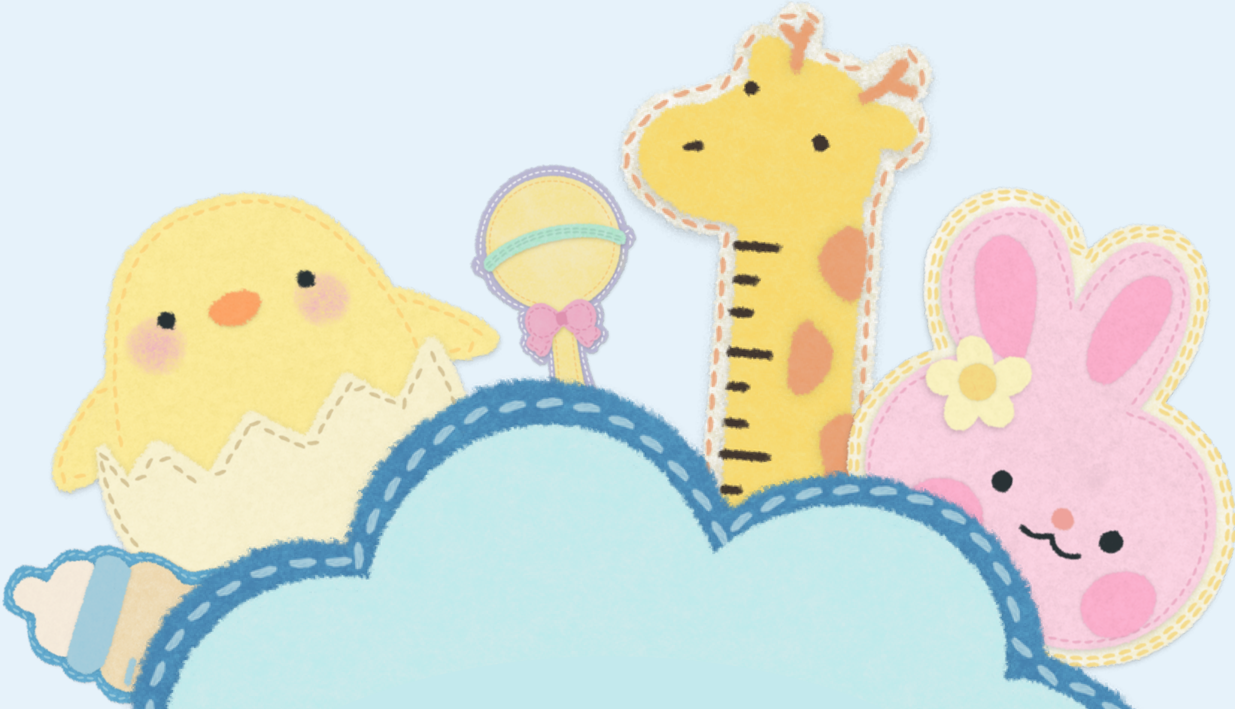

The Baby Grow Study

Instructions for Parents

Recording your baby's first year

SIMONS FOUNDATION

UNIVERSITY OF SUSSEX

Dear parent, a big congratulations from the Baby Grow team on the birth of your baby!

Thank you for choosing to become a citizen scientist alongside your new arrival. We're really happy that you're joining us as we undertake this important research into how infants' movement patterns interact with their social and communication skills in the first 18 months of life.

Our everyday physical actions like picking up a toy or copying a parent’s facial expressions are more than simple muscle responses. They're actually complex sequences of movements that are organised in a particular pattern. Development of motor (movement) skills during infancy occurs slightly before social and communication skills such as talking, and it’s thought that the two skills are related. However, the relationship is still unclear, so we're excited to track your baby’s movements and learn how infants’ motor and social and communication skills interact over time.

To do this, we will use wearable technology (onesies with movement sensors) and short videos to capture your child’s natural movements from 0 to 8 months. Some participants will just take videos, while others will collect data using both sensor-suits and videos.

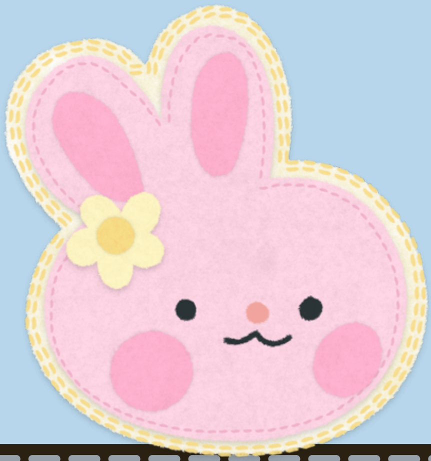

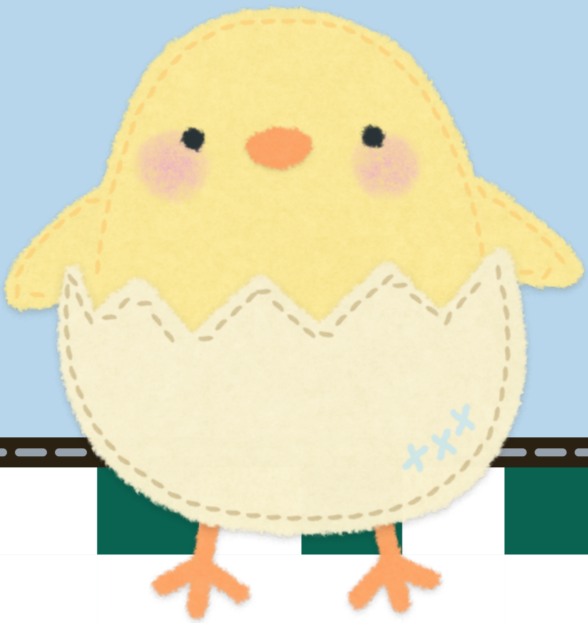

We will also be tracking the development of your child's motor and social-communication skills by asking you some questions about what they're doing twice a month.

When they are a month old, we will also ask you to do our Baby Grow "exercises" with your child while you're taking the video. We'll ask you to practice grasping with a silicone baby spoon that we've included in your kit, tummy time, pulling to sit and supported standing.

We believe this study may be vital in providing critical insights into earlier identification of children at risk of neurodevelopmental disorders like Autism Spectrum Condition (ASC), so parents and their babies who take part in this study will be giving us potential tools to recognise these conditions earlier in childhood, which would help many families get help and support for their child's development when they need it most.

Contents of the Baby Grow parent pack

All equipment required to complete a session will be located inside the Baby Grow parent pack provided by the research team. Below is the list of items each parent should receive either in the post or by home visit:

- The Baby Grow tote bag
- A yoga mat
- Two Baby Grow suits (size newborn and 0-3 months)
- A phone clamp
- A baby spoon

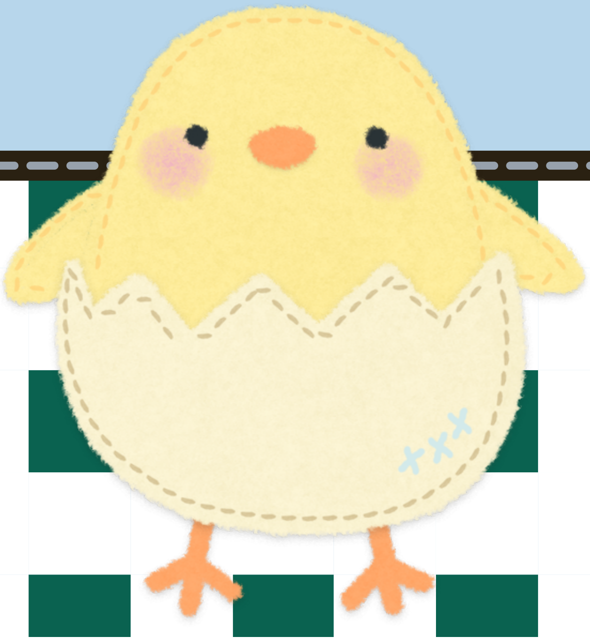

Tips for preparing your baby for the session.

- Make sure your phone is fully charged to collect the video data and your WIFI is stable.

- Your child should be awake and settled, and not crying or sucking a dummy during the video (babies move differently when they cry or suck).

- Parents should choose to record the session at the time of day when their child is most content (e.g., after a feed, or a time when they are alert and settled).

- If your child is unsettled on the day you are due to record a session, you can attempt another session on a different day but please ensure sessions are a minimum of 6 days apart.

Setting up the video collection experiment

1.Experimental session

Equipment needed:

- The yoga mat
- The provided suit
- The phone clamp

1.The yoga mat provided should be laid out on a flat surface in a well-lit room with no visible distractions (i.e., T.V, toys, pets) with the rulers facing up. The short side ruler will be the end the baby's feet will be.

2.The phone clamp should now be securely attached to an appropriate surface (i.e., high enough for the camera to capture the suit and all limbs when lying on the mat and at least one of the measuring tapes). Put the phone in the clamp with the screen facing up and the rear camera facing down so the parent can operate the phone while the camera Is pointing down. The video needs to be recorded from above the feet end of the child, and include the entire body in the image, as shown in the example below.

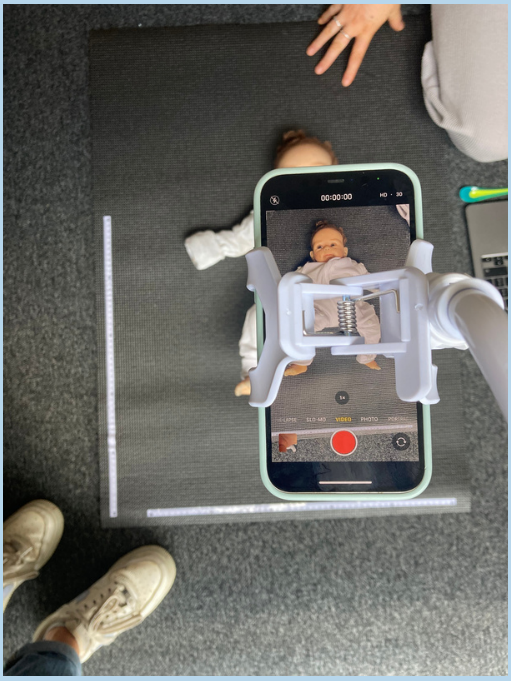

3.As soon as the equipment is ready, the child can be placed in the suit and placed on the yoga mat under the phone camera in the correct position.

2. Recording a session

1. Now the video recording can begin by pressing start on the phone camera. You should position yourself in a place that won’t affect the infant’s concentration (i.e., behind them).

2.Your child should be allowed to move spontaneously by themselves during the video with very limited interaction during the session (infants moves less when concentrating on adult interaction or watching a toy).

3.You should aim to record your baby moving naturally for about 4 minutes or until they get fussy or upset laying down. When they reach one month of age, please start doing the Baby Grow Exercises with your baby.

3. The Baby Grow Exercises

1.There are 4 exercises that we would like you to do with your baby, which you can start after 4 minutes of video or when your baby gets fussy.

2.It's really important that we can see all your baby's limbs when you do the exercises, so please have a look at the videos and pictures for tips on how to angle the phone camera to get the clearest shot, and always remain positioned behind your baby.

3.The first exercise (grasping the spoon), need your camera to be placed at the same overhead angle used to capture all your baby’s arms, legs and head movements in the usual video recording. The rest of the exercises (sitting, standing and tummy time), will require you to use a tilted, low-level angle, and for the sitting and tummy time exercises, your baby will need to be side on, not facing the camera - please see the photos in this document and our YouTube video for more guidance.

The Baby Grow Exercises YouTube Video

Holding an object:

1. Pick up the spoon provided in your kit by the handle. Move it slowly into the infant's view from the right side of their body (image 1), then place the spoon into your baby's hand so that they can hold it (image 2), but don't worry if your baby doesn't grasp it. Then repeat this from the left side. This is so we can see how they follow and hold the spoon.

2. Next, hold the spoon in the middle of your baby's body about 10 cm away from their chest or at a distance where they could reach out and grasp it (image 2). Wait for around 10 seconds or until your child reaches out to hold the spoon. If they grasp the spoon, wait a maximum of 1 minute or until the child releases it. Please remove the spoon from your child if they hurt themselves with it in any way! Don't worry if they don't grasp the spoon, you can move on to the next exercise.

Image 1:

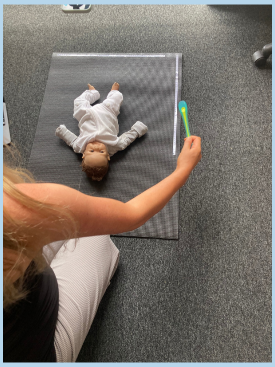

Image 2:

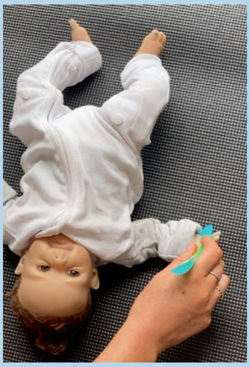

Image 3:

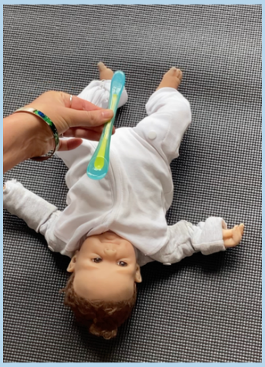

Changing your camera angle

For the next three exercises (sitting, standing and tummy time), you'll have to adjust your camera angle so it's at a low, 45 degree angle and your baby's head, arms and legs can be seen in the screen as shown in the images below and in our YouTube video.

For the **sitting and tummy time** tasks, your baby will need to be **side-on** the the phone camera, while for the **standing** task, they'll need to be **facing** the camera.

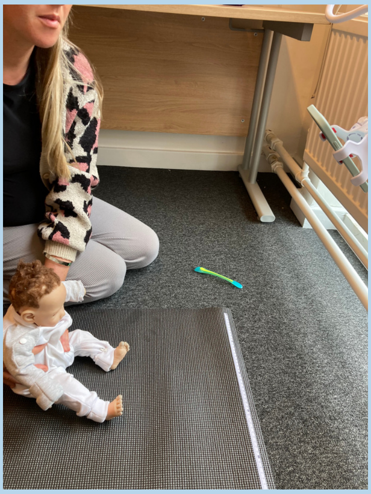

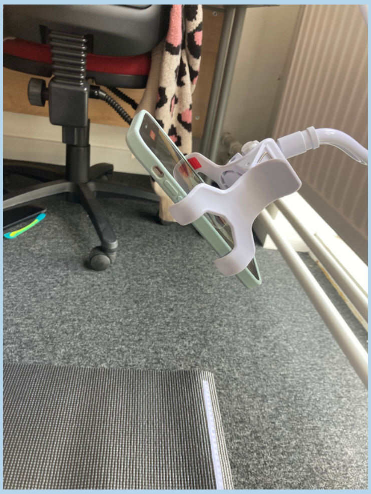

Sitting:

This task will change and progress when your baby shows they're stable in their current seated position. Our researchers will look at your baby's posture each week and ask you to change your baby's position when they are able to maintain their current position. All the sitting stages should be shown **side-on** to the camera

1. Supported sit:

Lift your baby from their laying position by placing your hands around their waist, supporting their neck if needed, and lift them to a fully supported sitting position (see image 4 and our YouTube video) with their side to the camera. If your baby's head lolls forward at any point or their back becomes rounded, gently lay your baby back down. If they are able to keep their head up and their back stays straight, you can keep them in a supported sitting position for a maximum of 1 minute. When they are able to maintain the supported sit for 1 minute, you can try the partially supported sit at your next video session.

Image 4:

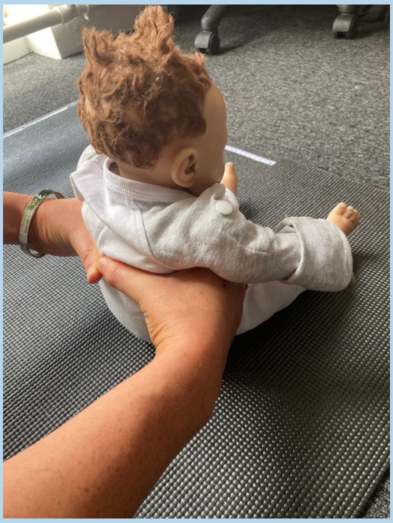

2. Partially supported sit:

Again, place your hands around the infant's waist, supporting the neck if needed, and lift them to a partially supported sitting position (see image 5 and video) by holding just their arms. Hold this position for 1 minute, if possible. When they are able to hold this position for 1 minute, you can try the next sitting position at your next session.

Image 5:

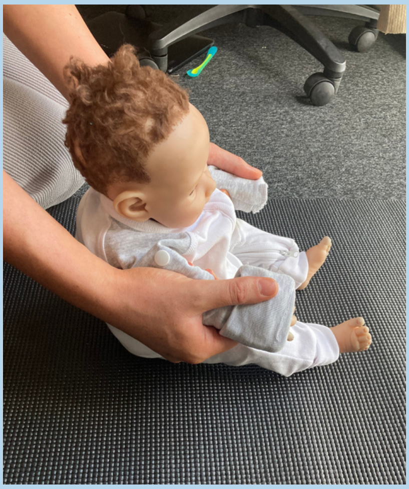

3. Sitting with arm support:

Once your baby can fully control their neck and can stay in a partially supported sit for 1 minute, place them in a seated position on the mat, by putting their arms in front of them so they can support their weight using their arms (see image 6). Infants can be left in this position for a maximum of one minute. Once your baby has mastered this position by maintaining it for 1 minute, you can move on to the unsupported sit at your next session.

Image 6:

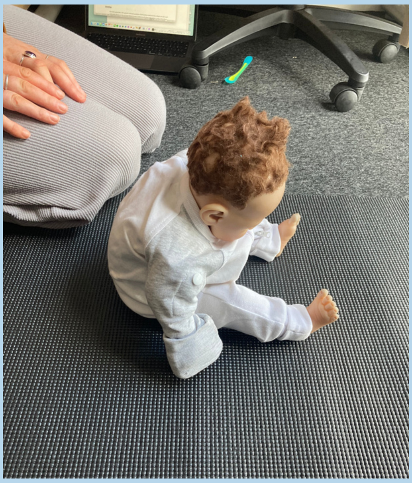

4. Unsupported sitting:

When your baby has mastered sitting with arm support, they should naturally move into an unsupported sit, where you can place your child in a seated position on the mat for a maximum of 1 minute (see image 7 below).

Image 7:

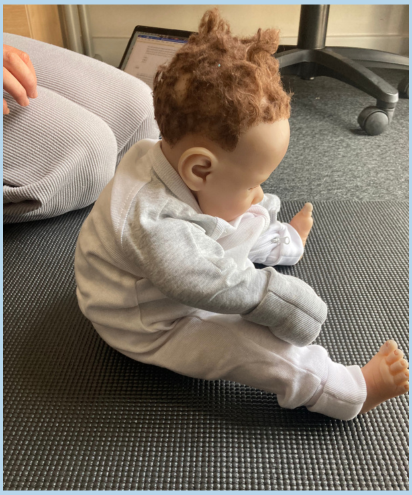

Standing:

For this exercise, from the sitting position (no matter what phase they're in), pick them up by placing your hands underneath their shoulders and under their arms, while still supporting their neck with your thumbs if needed (see image 8), and pull your baby to a standing position with their feet on the mat. Your baby should be **facing** the camera. This exercise should be performed until your baby gets fussy, looks uncomfortable or for a maximum of 1 minute. See image 8 below.

Image 8:

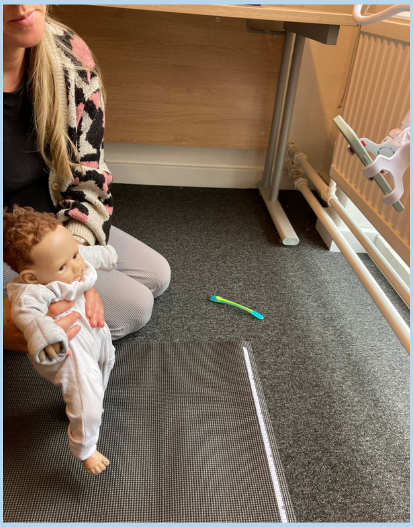

Tummy Time

Please watch the additional YouTube video link for tummy time guidance before completing this task

Tummy Time

1. Keeping your camera angle at the same 45 degree angle, gently place your baby on their tummy **with their side to the camera** and their hands next to their face (see image 9 below). We understand that lots of babies get fussy quickly during tummy time, so if they seem upset at any point, stop and move on to the next exercise - otherwise you can leave your baby in this position for a maximum of 1 minute, before moving on to the next exercise.

Image 9:

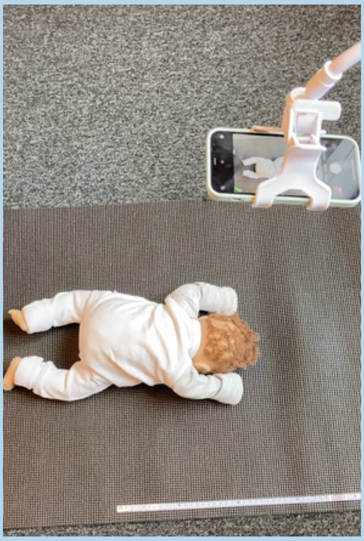

4. Ending a session

1.Depending on the age of your baby, the video recording can be stopped after 5 minutes (newborn to 1 month of age) or about 8-10 minutes (if they are 1 month or older and you are doing the Baby Grow Exercises with them).

2.The suit can then be removed, and all equipment stored in the Baby Grow bag provided to ensure it is always kept safe and out of reach from any children.

5. Sending the data

1.The recorded video should be sent via WeTransfer to: **h.j.rowan@sussex.ac.uk**.

2.The video should be titled as the date of the session (e.g., 06/08/2023), then select next. Then select ‘send an email’.

3.A team member will contact you if there are any problems with viewing the video or it hasn’t been received.

Troubleshooting and FAQ

Sensor Suit:
Q. What if the suit gets dirty?
A. Please don’t worry about the suit getting dirty, just remove all the sensors, store them in their box and machine wash the suit as normal.

Q. What if the suit gets damaged?
A. We are happy to replace and repair any damaged suit if necessary, please make the team aware as soon as possible.

Your child:
Q. What if either myself or my child is unwell?
A. If you or your child become unwell, please contact a member of our research team so we can make a note that data collection will be on hold until you're able to start the study again.

Q. What if the video/session only lasted 2 minutes?
A. Please don't worry if the video is short, the data will still be very valuable so please send the session over to us!

Equipment:
Q. I’ve lost something!
A. Please let us know if anything has been misplaced and we will send or bring you a replacement.
